# Deciphering gamma-decalactone biosynthesis in strawberry fruit using a combination of genetic mapping, RNA-Seq and eQTL analyses

**DOI:** 10.1186/1471-2164-15-218

**Published:** 2014-04-17

**Authors:** José F Sánchez-Sevilla, Eduardo Cruz-Rus, Victoriano Valpuesta, Miguel A Botella, Iraida Amaya

**Affiliations:** 1 Instituto Andaluz de Investigación y Formación Agraria y Pesquera, IFAPA-Centro de Churriana, Cortijo de la Cruz s/n, 29140 Málaga, Spain; 2 Departamento de Biología Molecular y Bioquímica, Instituto de Hortofruticultura Subtropical y Mediterránea (IHSM-CSIC-UMA), 29071 Málaga, Spain; 3 Horticultural Sciences Department, University of Florida, 1301 Fifield Hall, Gainesville, FL 32611, USA

**Keywords:** Aroma, Crop improvement, Desaturase, Flavor, Hydroxylase, Lactone, eQTL

## Abstract

**Background:**

Understanding the basis for volatile organic compound (VOC) biosynthesis and regulation is of great importance for the genetic improvement of fruit flavor. Lactones constitute an essential group of fatty acid-derived VOCs conferring peach-like aroma to a number of fruits including peach, plum, pineapple and strawberry. Early studies on lactone biosynthesis suggest that several enzymatic pathways could be responsible for the diversity of lactones, but detailed information on them remained elusive. In this study, we have integrated genetic mapping and genome-wide transcriptome analysis to investigate the molecular basis of natural variation in γ-decalactone content in strawberry fruit.

**Results:**

As a result, the fatty acid desaturase *FaFAD1* was identified as the gene underlying the *locus* at LGIII-2 that controls γ-decalactone production in ripening fruit. The *FaFAD1* gene is specifically expressed in ripe fruits and its expression fully correlates with the presence of γ-decalactone in all 95 individuals of the mapping population. In addition, we show that the level of expression of *FaFAH1*, with similarity to cytochrome p450 hydroxylases, significantly correlates with the content of γ-decalactone in the mapping population. The analysis of expression quantitative trait loci (eQTL) suggests that the product of this gene also has a regulatory role in the biosynthetic pathway of lactones.

**Conclusions:**

Altogether, this study provides mechanistic information of how the production of γ-decalactone is naturally controlled in strawberry, and proposes enzymatic activities necessary for the formation of this VOC in plants.

## Background

The flavor and aroma of strawberries (*Fragaria × ananassa*) arise from a specific combination of sugars, acids and volatile organic compounds (VOCs) that varies widely among different cultivars and *Fragaria* species [1]. More than 360 VOCs have been detected in strawberry, including esters, aldehydes, ketones, alcohols, terpenes, furanones, and sulfur compounds [2-6]. Lactones constitute a group of fatty acid-derived flavor molecules, which have γ-(4-) or δ-(5-)-lactone structures, and have been isolated from bacterial, plants and animal sources [7,8]. Fruits are considered as a particularly rich source of lactones, conferring peach-like aroma and flavor in order to attract feeders for seed dispersal [9,10]. During strawberry maturation, the levels of compounds defined as green-volatiles decrease whereas levels of flavor compounds characteristic of ripe fruits, including esters and lactones, increase in parallel to other ripening-regulated processes such as anthocyanin accumulation [4]. Up to 10 different lactones have been identified in strawberry [1,3,11] and, among them, γ-decalactone is the most abundant, reaching maximum levels in fully red ripe fruits [4,12].

Lactones containing 8–12 carbon atoms are very potent flavor constituents in a variety of fruits such as strawberry, pineapple and peach. Biosynthetic studies indicate that several pathways originating from β-oxidation of unsaturated fatty acids are responsible for the structural diversity of lactones [9,10,13]. All lactones originate from their corresponding 4- or 5-hydroxy carboxylic acids, although the precise mechanism by which these substrates are produced remains elusive [9]. However, four different mechanisms have been proposed, suggesting that the oxygen atom could be introduced by either (1) reduction of oxo acids by NAD-linked reductases, (2) hydration of unsaturated fatty acids, (3) epoxidation and hydrolysis of unsaturated fatty acids, or (4) reduction of hydroperoxides [9,14]. To the best our knowledge, the enzymes specifically involved in the formation of lactones have not yet been reported, however, candidate enzymatic activities, such as acyl-CoA dehydrogenase, which is the first enzyme in fatty acid β-oxidation, have been proposed to be important for lactone production [15]. Epoxide hydrolases have been associated to γ-dodecalactone biosynthesis in peach, implying that the synthesis of this lactone could proceed through epoxidation of unsaturated fatty acids [10]. Alternatively, the hydroxylation of unsaturated fatty acids could involve desaturases and cytochromes P450 (CYP) or other hydroxylases not related to CYPs [14].

The concentration of lactones in peach is controlled by multiple *loci* with quantitative effects, quantitative trait loci (QTL), hampering the identification of the genetic determinants controlling their biosynthesis or regulation [16]. In contrast, the content of γ-decalactone has been shown to be controlled by one dominant *locus* in strawberry and, consequently, a number of strawberry cultivars lacking γ-decalactone have been reported [5,6,17]. We have previously reported that γ-decalactone is produced in the parental line ‘1392’ but not in ‘232’ of a strawberry mapping population and that the segregation of their F1 progeny matched a Mendelian 1:1 ratio with the *locus* controlling this trait mapped to the bottom arm of LG III-2 [6]. In this context, this population represents a valuable tool to identify the gene(s) responsible for the natural variation of this VOC in strawberry, and to provide novel information about its biosynthesis in plants.

Although cultivated strawberry is an octoploid (2n = 8× = 56), and as such, four subgenomes are present in its complex genome, most *loci* show a disomic segregation. Furthermore its genome has a high level of conservation with the model species *Fragaria vesca* (2n = 2× = 14), including an almost complete synteny and high colinearity [18-22]. Thus, the available genome sequence of the diploid *F. vesca* can be used as a reference for genomic and genetic studies within the genus [23].

RNA-seq is replacing other methods of quantifying transcript expression, including microarray platforms [24], as it overcomes some of their limitations, such as detection of only those transcripts that are represented on microarrays, low dynamic range (limited upper and lower limits of detection), and thus provides more accurate quantification of differential transcript expression. A clear advantage of RNA-seq is the detection of novel non-annotated transcripts and, most relevant for highly heterozygous plants and polyploids such as *F. × ananassa,* the detection of the different alleles and homoeologous genes within their genomes [24,25]. In this report, we have combined genome-wide RNA-seq analysis to a bulk segregant approach to identify a gene controlling γ-decalactone content in strawberry. Additional candidate genes of the biosynthetic pathway of lactones are also reported based on this genome-wide analysis. All together, this study provides information of how the content of γ-decalactone is naturally controlled in strawberry fruit and proposes enzymatic activities necessary for the formation of this VOC in plants.

## Methods

### Plant material

The ‘232’ × ‘1392’ F1 mapping population, comprising 95 progeny lines, was used in this study. This population is derived from the cross between selection lines ‘232’ and ‘1392’ and is described in detail in [19]. ‘232’ is a very productive strawberry (*Fragaria × ananassa*) line, whereas ‘1392’ has firmer and tastier fruits [6,19]. The mapping population was grown in the strawberry-producing area of Huelva (Spain) under commercial conditions during the 2011/2012 season. Six plants of each line were vegetatively propagated and grown. Ripe fruits (10-15) were collected the same day from the six plants of each line, divided into three biological replicates and independently grinded in liquid nitrogen. Samples were stored at -80ºC until further analysis.

### RNA isolation and RNA-seq from pooled samples

Equivalent amounts of ripe fruit tissue from 10 γ-decalactone producing and non-producing progeny lines (Table 1) were collected in triplicate and separately pooled for RNA extraction. The three biological replicates of each bulked pool were named H γ-DEC1-3 and N γ-DEC1-3 (for High and No γ-decalactone pool, respectively) and used in the analysis. Total RNA was extracted from pooled strawberry fruits using a differential 2-butoxyethanol precipitation-based method [26]. Prior to reverse transcription, RNA was treated with DNase I (Fermentas) to remove contaminating genomic DNA. RNA quantity and quality were determined based on absorbance ratios at 260 nm/280 nm and 260 nm/230 nm using a Nanodrop. RNA integrity was confirmed by the appearance of ribosomal RNA bands and lack of degradation products after separation in agarose gel electrophoresis and ethidium bromide staining. The integrity of the RNA samples was further verified using the 2100 Bioanalyzer (Agilent, Folsom, CA) and RIN values ranged between 7.2 and 7.4 for the six samples.

**Table 1:** Relative concentration of γ-decalactone in fruits of selected progeny lines producing and not producing this compound

**Fruit samples with γ-decalactone**					**Fruits w/o γ-decalactone**		
**Line**	**2007**	**2008**	**2009**	**Line**	**2007**	**2008**	**2009**
**93-01**	4.456	1.287	2.849	**93-03**	0.000	0.019	0.014
**93-12**	3.114	1.185	1.875	**93-07**	0.002	0.001	0.005
**93-19**	3.101	4.300	2.865	**93-14**	0.010	0.007	0.019
**93-36**	3.022	3.884	3.182	**93-17**	0.001	0.003	0.002
**93-43**	4.237	3.394	3.074	**93-18**	0.001	0.008	0.006
**93-54**	3.675	3.376	3.902	**93-49**	0.009	0.007	0.010
**93-61**	2.403	1.511	3.293	**93-68**	0.005	0.001	0.040
**93-64**	3.413	1.117	2.878	**93-69**	0.000	0.004	0.007
**93-78**	1.926	1.171	2.644	**93-80**	0.005	0.014	0.013
**93-92**	2.094	1.457	2.658	**93-89**	0.002	0.004	0.008

Content in each line is expressed as a ratio relative to the content of γ-decalactone in a reference sample containing a mix of all mapping lines for each year.

For each of the 6 (2 bulks with 3 biological replicates) samples, one paired-end library with approximately 300 bp insert size was prepared using an in-house optimized Illumina protocol at the Centro Nacional de Análisis Genómico (CNAG) facilities. Libraries were sequenced on Illumina HiSeq2000 lanes using 2 × 100 bp reads. More than 30 million reads were generated for each sample. Primary analysis of the data included base calling and quality control, with an assurance that >80% of all bases passing filter had a quality value of at least 30.

### Mapping RNA-seq reads to the reference genome and generation of read counts

Raw RNA-seq reads were processed to remove low-quality nucleotides and aligned to the *Fragaria vesca* reference genome (v1.1) and CDS (v1.0) [23] using the program TopHat v2.0.6 [27]. Default parameters of TopHat were used, allowing 40 multiple alignments per read and a maximum of 2 mismatches when mapping reads to the reference. The mapping results were then used to identify “islands” of expression, which can be interpreted as potential exons. TopHat builds a database of potential splice junctions and confirms these by comparing the previously unmapped reads against the database of putative junctions.

The aligned read files were processed by Cufflinks v2.0.2 [28]. Reads were assembled into transcripts, their abundance estimated, and tests for differential expression and regulation between the samples were performed. Cufflinks does not make use of existing gene annotations during assembly of transcripts, but rather assembles a minimum set of transcripts that best describe the reads in the dataset. This approach allows Cufflinks to identify alternative transcription and splicing that are not described by pre-existing gene models [28]. The normalized RNA-seq fragment counts were used to measure the relative abundances of transcripts expressed as Fragments Per Kilobase of exon per Million fragments mapped (FPKM). Confidence intervals for FPKM estimates were calculated using a Bayesian inference method [28].

### Comparison to reference annotation and differential expression analysis

Once all short read sequences were assembled with Cufflinks, the output GTF files were sent to Cuffcompare along with a reference GTF annotation file, downloaded from Genome Database for Rosaceae (GDR) database (*Fragaria vesca* Whole Genome v1.1 Assembly & Annotation. http://www.rosaceae.org/). This classified each transcript as known or novel. Cuffcompare produced a *combined. GTF* file which was passed to Cuffdiff along with the original alignment (.SAM) files produced by TopHat to identify differentially expressed transcripts between the two pools. The Cuffdiff algorithm then re-estimated the abundance of transcripts listed in the GTF file using alignments from the SAM file, and concurrently tested for differential expression between the high γ-decalactone and the no γ-decalactone pools using a rigorous statistical analysis [28]. The significance scores were corrected for multiple testing using the Benjamini-Hochberg correction. The expression testing is done at the level of transcripts, primary transcripts and genes. By tracking changes in the relative abundance of transcripts with a common transcription start site, Cuffdiff can also identify changes in splicing.

### Visualization of mapped reads

Mapping results were visualized using a local copy of the Integrative Genomics Viewer software available at http://www.broadinstitute.org/igv/. Views of individual genes were generated by uploading TopHat-generated files containing the sequence alignment data (.bam files) to the genome browser.

### Functional analysis of gene lists using BLAST2GO

The BLAST2GO v 2.4 suite was used for functional annotation of sequences, data mining and gene set enrichment analysis [29]. The functional clustering tool was used to look for functional enrichment for genes over- and under-expressed more than two-fold between the pools. GO enrichment was derived with Fisher’s exact test and a cutoff of false discovery rate < 0.05 using the *F. vesca* genome annotation as reference background. A unique list of gene symbols was uploaded via the web interface. Gene Ontology Biological Process was selected as the functional annotation category for this analysis.

### *De novo* assembly of *Fragaria × ananassa* RNA-seq reads

Since the current *F. vesca* genome sequence and the gene model is still a draft, some RNA-seq transcript sequences appeared truncated. Therefore, we proceeded to a *de-novo* assembly of the reads corresponding to the high- and no-γ-decalactone pools to obtain the full-length transcripts expressed in *F. × ananassa* using *Trinity*[30]. The transcript contigs most similar to the *F. vesca* candidate genes were identified by mean of blast search.

### Sequence analysis

Multiple sequence alignment was carried out with CLUSTALW at the default settings. Phylogenetic analyses were conducted using the neighbor-joining algorithm and Poisson model in MEGA version 5 [31]. Protein targeting predictions were done using WoLF PSORT analysis (http://wolfpsort.org) and transmembrane domain search with the TMpred program (http://www.ch.embnet.org/software/TMPRED_form.html).

### Real time qRT-PCR analysis

Total RNA was extracted from strawberry tissues as described previously for the RNA-seq experiment. First-strand cDNA was synthesized from 1 μg of total RNA using the iScript cDNA Synthesis kit (Bio-Rad) according to the manufacturer’s instructions. Gene expression was analyzed by quantitative real time polymerase chain reaction (qRT-PCR) using the fluorescent intercalating dye SYBRGreen I in an iQ5 real-time PCR detection system (Bio-Rad). Three biological replicates for each line and three independent synthesis of cDNA for each RNA sample were used for qRT-PCR. Relative quantification of the expression levels for the target genes was performed using the comparative Ct method [32]. Glyceraldehyde-3-phosphate dehydrogenase gene (*GAPDH*) was used as normalizing gene [33]. Primers are described in Additional file 1: Table S1.

### QTL and expression quantitative trait loci (eQTL) analysis

QTL analyses were performed using MapQTL 5 as previously described [6]. The raw relative data was analyzed first by the nonparametric Kruskal-Wallis rank-sum test. A stringent significance level of P = 0.005 was used as threshold. Second, the integrated genetic linkage map and transformed data sets for most traits were used to identify and locate QTLs using Interval Mapping. Significance LOD thresholds were estimated with a 1,000-permutation test for each trait and QTLs with LOD scores greater than the genome-wide threshold at 95% were declared significant.

## Results

**Figure 1:**
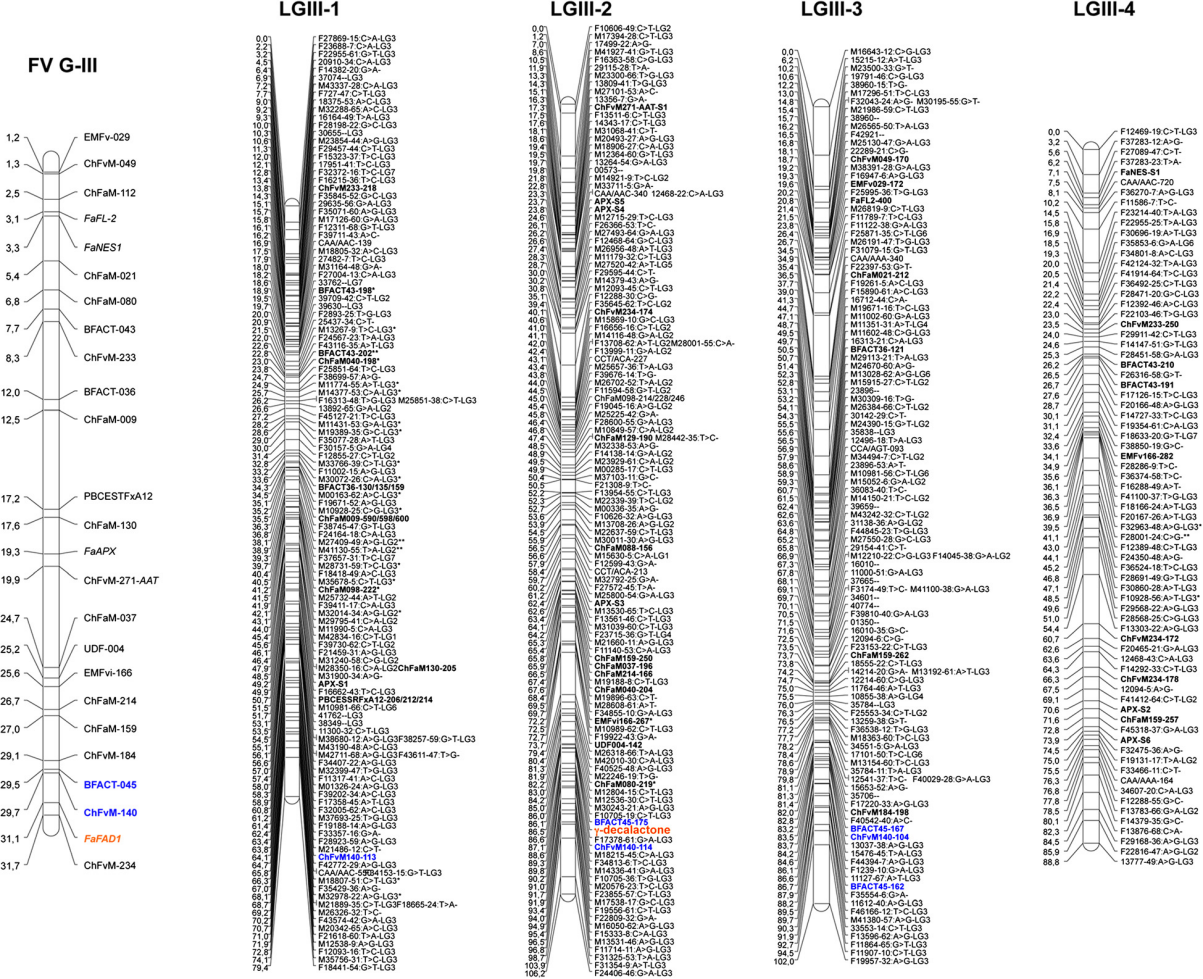
**Comparison of pseudochromosome 3 (FV G-III) of the diploid *Fragaria vesca *with the four homoeologous linkage groups on the integrated ‘232’ × ‘1392’ linkage map (LGIII-1–LGIII-4) of the octoploid *F. x ananassa*.** The γ-decalactone locus is highlighted in orange and linked SSR markers in blue. SSR and gene markers are highlighted in bold. Position of markers (in cM) is indicated on the left of the linkage groups. For simplicity, only the position of anchor SSR markers (in Mb) is stated on the left of the *F. vesca* group.

In order to increase the precision of the ‘232’ × ‘1392’ map and identify new markers closely linked to the *locus* controlling γ-decalactone, we first saturated the previous map with DArT-derived SNP markers and developed a saturated map. The octoploid strawberry homoeology group (HG) III, where the *locus* was previously mapped [6], is presented in Figure 1 in comparison to the *F. vesca* pseudochromosome 3 (fvesca_v1.1_pseudo.fna). The 4 homoelogous linkage groups (LGs), with lengths of 79.4, 106.2, 102 and 88.8 cM, could now be identified instead of 7 shorter LGs in the previous integrated map [6]. The average marker density in HG III was increased to 0.87 cM/marker and the largest gap ranged from 5.3 cM for LG III-1 to 7.7 cM for LG III-3 (Figure 1; Additional file 2). The locus controlling γ-decalactone was fine-mapped to the bottom of *Fragaria × ananassa* LG III-2, closely linked to markers BFACT-45 and ChFvM140, consistent with our previous data [6]. In addition, six new SNP markers were mapped in the ± 3 cM interval to the γ-decalactone *locus* (Figure 1; Additional file 2).

In order to identify the determinants of the variation in γ-decalactone content in strawberry fruit, we aimed to identify differentially expressed genes between pools of fruits from lines contrasting in γ-decalactone content in the ‘232’ × ‘1392’ population using RNA-seq. Later, differentially expressed genes would be analyzed for their mapping position. Those genes convening the two conditions, i.e., highly expressed in fruits of high γ-decalactone lines and located within the QTL interval would be considered for further analysis. RNA was extracted from bulked pools of ripe fruits from 10 progeny lines with high γ-decalactone content and from 10 lines not producing the volatile (Table 1) and used in biological triplicate for Illumina RNA sequencing. An alignment of sequencing reads was performed using the reference *Fragaria vesca* Whole Genome (v1.1) and annotation (CDS v1.0) [[23]; Genome Database for Rosaceae (GDR), http://www.rosaceae.org] using TopHat [27]. Over 218 million reads 100 bp-long were generated and after removal of adaptor sequences and low-quality reads, 211.6 million clean reads remained (97% of the raw data). Between 68.7% and 70% of reads were paired for each of the 6 samples and an average of 68.2% of filtered paired reads were further mapped to the *F. vesca* genome. Some key metrics that allowed the assessment of the quality of mapping reads to the reference genome were extracted from the TopHat output and log files, and are shown in Additional file 1: Table S2.

After mapping the RNA-seq reads to the reference genome, transcripts were assembled and their relative abundances calculated using Cufflinks [28]. Genes with normalized reads lower than 0.1 fragments per kilobase of exon per million fragments (FPKM) were considered as not expressed. A total of 33,458 gene/transcripts from the two *F. x ananassa* pools were predicted based in the reference model and 19,833 and 19,720 were expressed in the ripe fruits of the high γ-decalactone and the no γ-decalactone pools, respectively.

Differential gene expression (DGE) between the high γ-decalactone and the no γ-decalactone pools was calculated using the ratio of FPKM values of each gene in both pools. A total of 617 predicted genes were differentially expressed between the two pools and re-annotated using *Blast2go*[29]. Of these, 403 were up-regulated and 214 were down-regulated in the high-γ-decalactone pool (Additional file 3). The observed ratios (log2 fold change) of differential expression ranged from -5.161 to 3.489, with negative and positive values indicating up- and down-regulation in the high-γ-decalactone pool, respectively. Only one gene (gene24970-v1.0-hybrid) encoding for a predicted protein with similarity to cinnamyl alcohol dehydrogenase, was not expressed at all in the no-γ-decalactone pool and expressed in the high-γ-decalactone pool, albeit with a relatively low value of expression (3.79 FPKM). Among the 617 differentially expressed transcripts, 577 corresponded to annotated genes in the *F. vesca* gene model v.1.0 [23] while 40 matched with not annotated genome regions*.* Among these, 28 corresponded to predicted genes from *F. vesca* recently annotated in the NCBI, while the remaining 12 transcripts have not yet been annotated. Some gene families appeared over-represented in the fruits with high γ-decalactone content such as cinnamyl alcohol dehydrogenases, with 6 differentially expressed genes, glutathione s-transferases, with 7 up-regulated genes, and cytochrome p450, with 5 up-regulated members (Additional file 3).

### Functional annotation and enrichment analysis

In order to describe gene functions in a standard and controlled vocabulary, we used the *Blast2GO* suite. A total of 3,757 gene ontology (GO) terms were assigned to a total of 559 differentially expressed genes, while 58 did not match any terms. Sequence distribution (at level 2, filtered by a cut-off of 60 sequences) for biological processes, molecular functions and cellular component are summarized in Additional file 1: Figure S1. Within biological processes, the most abundant categories were metabolic process (380 sequences or 27%), cellular process (350 sequences; 25%) and response to stimulus (210 sequences; 15%). The most represented molecular function was catalytic activity (299 sequences; 49%) and a large proportion of sequences (177; 22%) were associated with membrane as cellular compartment.

To investigate the biological processes associated with differences in γ-decalactone content, a GO enrichment analysis was performed using Fisher’s exact test using the sets of up-regulated and down-regulated transcripts separately in comparison to those in the reference *F. vesca* gene model. A total of 51 biological processes were significantly enriched for the genes up-regulated in fruits with high γ-decalactone content (Additional file 1: Table S3). Most of these 51 common ontologies are ‘descendants’ of 5 higher hierarchical nodes in the GO tree: response to stimulus (GO:0050896), cellular aromatic compound metabolic process (GO:0006725), lipid homeostasis (GO:0055088), nitrogen compound metabolic process (GO:0006807) and organic substance metabolic process (GO:0071704). Within these biological processes, the most significantly over represented term was oxidation-reduction process (GO:0055114, 92 genes within this term). One possible interpretation is that enzymes catalyzing the addition or removal of electrons are needed in the biosynthesis of γ-decalactone in strawberry.

The number of biological processes significantly enriched within the down-regulated genes (up-regulated in the no-γ-decalactone pool) was higher, 101, and more diverse (Additional file 1: Table S4). The three most significantly over represented terms were regulation of biological quality (GO:0065008), response to biotic stimulus (GOO:0009607) and ion transport (GO:0006811). Globally, these data suggest that lacking γ-decalactone in strawberry fruits is associated to a wide range of different biological processes. Particularly interesting is the number of genes up-regulated in the categories of response to stimulus and biotic stress in the absence of γ-decalactone.

### Identification of *FaFAD1* as the gene underlying the *locus* controlling γ-decalactone content in LG III-2

The top 25 up-regulated genes in the pool with high γ-decalactone content are listed in Table 2. The third gene with the highest up-regulation between the pools corresponds to the *Fragaria vesca* gene ID gene24414-v1.0-hybrid (gene24414), with homology to fatty acid desaturases (FAD) and in particular to the microsomal Δ-12 oleate desaturase (FAD2). This gene shows a high expression in the pool of fruits with γ-decalactone (325.92 FPKM) and its expression is ~30-fold higher (4.8 log2-fold) than in the pool of fruits without γ-decalactone (11.41 FPKM; Table 2; Additional file 3). Most interestingly, gene24414 maps to the end of the pseudo-molecule 3 of the *Fragaria vesca* reference genome, at the exact position where the gene controlling γ-decalactone has been mapped in *F. x ananassa* (Figure 1). In addition to gene24414, only 3 other genes among the 617 differentially expressed genes mapped to the γ-decalactone content *locus*. Two of them, gene24411 and gene24415, at about 34 and 9 Kb from gene24414, respectively, and a third, gene14386, separated by 808 Kb. However, the fold-change between the bulked pools for these 3 genes was much smaller than for gene24414, ranging from 0.5 to 0.9 log2-fold. Two of these genes, 24411 and 14386, with similarity to *callose synthase* and *glycerophosphoryl diester phosphodiesterase*, respectively, were down-regulated in the high γ-decalactone pool. The third gene, 24415 with highest similarity to the *nucleolar complex protein 2*, showed higher expression in the high γ-decalactone pool (Additional file 3).

**Table 2:** List of the top 25 significantly up-regulated genes in the high γ-decalactone pool compared to the No γ-decalactone pool

**Gene**	**Locus position**	**Predicted function**	**FPKM H γ-DEC**	**FPKM N γ-DEC**	**log2 fold change**	**Test statistic**	**p-value**	**q-value**
gene24970-v1.0-hybrid	LG1:16175288-16176946	Cinnamyl alcohol dehydrogenase-like	3.79	0.00	-1.8E + 308	-1.8E + 308	3.78E-15	2.06E-12
gene22145-v1.0-hybrid	LG4:24174473-24176874	Aldo keto reductase	1.98	0.06	-5.16	3.44	0.000573	0.021971
gene24414-v1.0-hybrid	LG3:31112417-31114643	Microsomal delta-12 oleate desaturase	325.92	11.41	-4.84	33.60	0	0
gene17831-v1.0-hybrid	LG1:12556715-12561321	Isoflavone 2 -hydroxylase-like	26.20	1.52	-4.10	13.80	0	0
gene11616-v1.0-hybrid	unanchored	---NA---	1.07	0.10	-3.49	6.49	8.34E-11	2.37E-08
gene12565-v1.0-hybrid	LG7:19409089-19409688	S-norcoclaurine synthase-like	8.52	1.00	-3.09	6.15	7.56E-10	1.61E-07
gene09812-v1.0-hybrid	LG6:9651860-9658187	Thaumatin-like protein	9.64	1.18	-3.03	8.53	0	0
gene29430-v1.0-hybrid	unanchored	Salicylic acid-binding protein 2-like	4.75	0.60	-2.98	4.62	3.92E-06	0.000362
gene09059-v1.0-hybrid	LG2:20948366-20950293	Hypothetical protein	1.10	0.14	-2.98	3.89	0.000102	0.005647
gene12485-v1.0-hybrid	LG1:6048496-6049132	Auxin-binding protein abp19a-like	2.15	0.28	-2.96	3.50	0.000468	0.018845
gene09427-v1.0-hybrid	LG5:10292998-10293780	Probable glutathione s-transferase-like	94.21	13.04	-2.85	17.49	0	0
gene16882-v1.0-hybrid	LG4:17649473-17650304	Probable glutathione s-transferase-like	5.13	0.76	-2.75	5.74	9.30E-09	1.60E-06
gene05671-v1.0-hybrid	LG6:28779898-28789864	Beta-glucanase	9.32	1.45	-2.69	10.13	0	0
-	LG6:25256895-25257433	Hypothetical protein	2.32	0.37	-2.67	3.24	0.001216	0.038671
gene08424-v1.0-hybrid	unanchored	Pathogenesis-related protein 4	7.04	1.15	-2.61	6.47	1.01E-10	2.79E-08
-	LG7:2164010-2164526	---NA---	4.04	0.67	-2.60	4.09	4.29E-05	0.002708
-	unanchored	---NA---	77.57	13.29	-2.55	3.37	0.000739	0.026493
gene13265-v1.0-hybrid	LG7:21691207-21692009	Par-1a protein	3.39	0.59	-2.53	4.10	4.17E-05	0.002653
-	LG4:2760209-2761669	Inhibitor of trypsin and hageman factor	4.34	0.76	-2.51	3.41	0.000656	0.024349
gene28799-v1.0-hybrid	LG3:9340369-9341925	Nectarin-3-like	2.84	0.52	-2.45	4.74	2.12E-06	0.000217
gene32603-v1.0-hybrid	LG4:2356895-2357929	Sieve element-occluding protein	1.82	0.34	-2.40	3.48	0.000503	0.019820
gene02395-v1.0-hybrid	LG3:5514062-5518181	Cytochrome p450	106.92	20.98	-2.35	19.15	0	0
gene21028-v1.0-hybrid	LG7:17655017-17664058	Psbp domain-containing protein chlorop-like	1.80	0.36	-2.33	4.37	1.24E-05	0.000952
gene17832-v1.0-hybrid	LG1:12556715-12561321	Hypothetical protein	19.76	4.11	-2.26	5.07	3.9E-07	4.78E-05
-	LG3:10391258-10391650	---NA---	14.68	3.06	-2.26	3.78	0.000156	0.007817

Gene number according to the *Fragaria vesca* genome draft (http://www.Strawberrygenome.org). For a number of transcripts detected by cufflinks (-), no reference transcript was predicted in the *Fragaria vesca* gene model.

All of the reads from the high γ-decalactone pool mapping to the gene24414-v1.0-hybrid (hereafter named *FaFAD1* and *FvFAD1* for the cultivated and wild strawberry, respectively) corresponded to one unique allele, indicating that only one allele is expressed in fruits of the 10 selected siblings. Similarly, all the reads from the no-γ-decalactone pool corresponded to the same allele to that in the fruits with high content.

**Figure 2:**
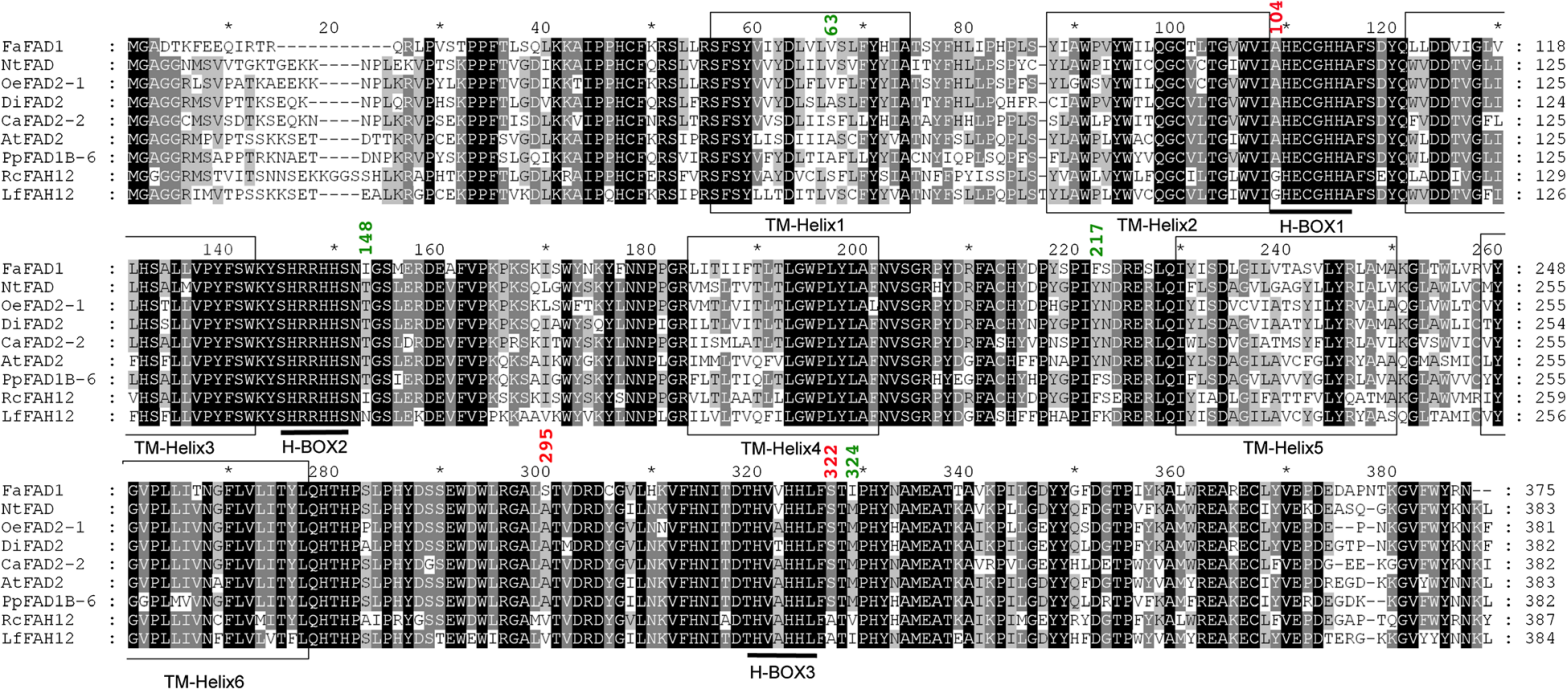
**Comparison of the amino acid sequence of strawberry FaFAD1 with other plant fatty acid desaturases (Δ12-FADs) and hydroxilases (Δ12-FAH).** Identical amino acid residues were indicated with black background. Dark and bright gray shade indicated 80% and 60% or more conservation among all the aligned sequences, respectively. The predicted trans-membrane domains (TM-helixes) and highly conserved His boxes are shown. The seven amino acid residues that differ between oleate desaturases and hydroxilases according to [45] (numbering based on the arabidopsis FAD2 sequence) are indicated in green when the amino acid residue is conserved between FaFAD1 and hydroxilases and red when not. Accession numbers for the sequences were as follows: *Fragaria x ananassa* FaFAD1 (KF887973), *Nicotiana tabacum* NtFAD (AAT72296), *Olea europaea* OeFAD2-1 (AAW63040), *Davidia involucrate* DiFAD2 (ABZ05022), *Crepis alpina* CaFAD2-2 (ABC00770), *Arabidopsis thaliana* AtFAD2 (AAA32782), *Prunus persica* PpFAD1B-6 (AGM53489), *Ricinus comunis* hydroxylase RcFAH12 (AAC49010), (MDP0000288297), *Lesquerella fendleri* bifunctional hydroxylase/desaturase LFAH12 (AAC32755).

The predicted gene24414 in the strawberry draft genome sequence [23] contains 5 exons. However, when visualized using the integrative genomics viewer (IGV), the reads of the RNA-seq experiments only spanned the first two exons indicating that *FvFAD1* was not correctly annotated in the strawberry genome sequence. A different model of the same gene that only spans the two first exons is available in the NCBI under accession number LOC101309231, and is predicted to encode a protein of 393 amino acids. However, the last 16 amino acids of the carboxylic end of the predicted protein do not have similarity to reported FAD2 proteins (data not shown). In order to unequivocally determine the *Fragaria x ananassa FAD1* transcript, we performed a *de novo* assembly of the RNA-seq reads from the high γ-decalactone pool replicates. Only one contig of 1847 bp with high similarity (excluding 25 bp of the 3′ end of the ORF and the 3′UTR) to the *F. vesca FvFAD1* gene was obtained, and contained an ORF of 1125 bp encoding a predicted protein of 375 amino acid residues. In this prediction, the C-terminus shares high similarity to FAD2 proteins except that FaFAD1 lacks the two last amino acids (Figure 2). However, the lack of these two amino acids is also found in the predicted amino acid sequence encoded by a *F. × ananassa* EST (accession number CO381851) available in the NCBI, indicating that this might be the correct sequence. The full length ORF of *FaFAD1*, including a 3′ un-translated region, was cloned from parental line ‘1392’ using primers deduced from the transcriptome assembly and confirmed the predicted sequence. In agreement to previous results using the reference *F. vesca* genome, the same *FaFAD1* allele was obtained after *de novo* assembly of the RNA-seq reads from the no-γ-decalactone pool.

The deduced FaFAD1 protein sequence contains the Delta12 Fatty Acid Desaturase (Delta12-FADS)-like conserved domain (E-value: 1.74e-56). Membrane FADs are non-heme, iron-containing, oxygen-dependent enzymes involved in regioselective introduction of double bonds in fatty acyl aliphatic chains. These enzymes are responsible for the synthesis of 18:2 fatty acids in the endoplasmic reticulum. Six putative transmembrane domains are predicted within FaFAD1 using the TMpred program as expected for an integral membrane protein (Figure 2). Alignment with other characterized FAD2 proteins indicated that the characteristic His-rich motifs, which contribute to the interaction with the electron donor cytochrome b5, were conserved in the deduced FaFAD1 protein. The most similar protein to FaFAD1 in Arabidopsis was the endoplasmid reticulum localized oleate desaturase FAD2 catalyzing the conversion of oleic acid (18:1) to linoleic acid (18:2) [34].

**Figure 3:**
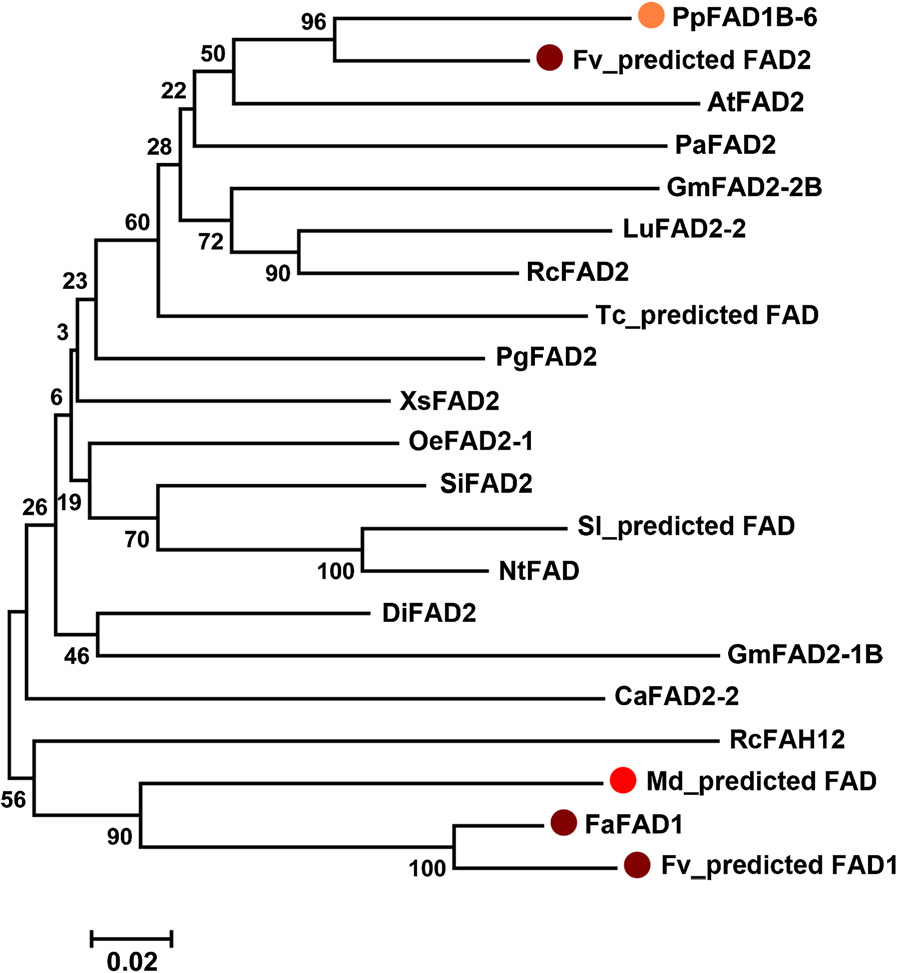
**Phylogenetic tree showing protein sequence relationships among selected FAD2 members from different species.** Bootstrap values (%) for 1000 replicates are indicated at the nodes. Position of the *Fragaria*, *Malus* and *Prunus* amino acid sequences is labeled in dark red, light red and orange, respectively. Accession numbers are shown in parenthesis: *Prunus persica* PpFAD1B-6 (AGM53489), *Fragaria vesca* Fv_predicted FAD (LOC101290788), *Arabidopsis thaliana* AtFAD2 (AAA32782), *Persea americana* PaFAD2 (AAL23676), *Glycine max* GmFAD2-2B (BAD89863), *Linum usitatissimum* LuFAD2-2 (ACF49507), *Ricinus communis* RcFAD2 (ABK59093), *Theobroma cacao* Tc_predicted FAD (EOY09487), *Punica granatum* PgFAD2 (AAO37754), *Xanthoceras sorbifolia* XsFAD2 (AGO32050), *Olea europaea* OeFAD2-1 (AAW63040), *Sesamum indicum* SiFAD2 (AAF80560), *Solanum lycopersicum* Sl_predicted FAD (XP_004228665), *Nicotiana tabacum* NtFAD (AAT72296), *Davidia involucrate* DiFAD2 (ABZ05022), *Glycine max* GmFAD2-1B (BAD89861), *Crepis alpina* CaFAD2-2 (ABC00770), *Ricinus comunis* RcFAH12 (AAC49010), *Malus domestica* Md_predicted FAD (MDP0000288297), *Fragaria x ananassa* FaFAD1 (KF887973) and *Fragaria vesca* Fv_predicted FAD1 (LOC101309231).

Highly similar proteins to FaFAD1 were identified after a blastp search in the NCBI and the phytozome database (http://www.phytozome.net). As shown in Figure 3, the phylogenetic analysis indicates the presence of two major clusters. As expected, the FaFAD1 protein was most similar to the *F. vesca* predicted protein encoded by the gene24414, located at the bottom of chromosome 3, and also to a predicted FAD protein from the closely related *Malus* genus. Interestingly, this group of protein sequences grouped with the *Ricinus communis* fatty acid hydroxylase RcFAH12. This protein, which shares high sequence similarity to desaturases, has been shown to catalyze the hydroxylation of oleate to produce the hydroxy fatty acid ricinoleate [35]. A recently identified FAD from *Prunus persica*, PpFAD1B-6, has been associated with lactone content in peach fruits using an integrative ‘omics’ approach [14]. This protein grouped in a second cluster with the *Ricinus communis* desaturase RcFAD2 and other reported desaturases, such as the Arabidopsis AtFAD2.

To further investigate whether the down-regulation of *FaFAD1* is the cause for the extremely low γ-decalactone content in strawberry fruits, we first validated the differential expression observed in the pools by qRT-PCR. As shown in Additional file 1: Figure S2A, the expression of *FaFAD1* was ~30-fold higher in the high-γ-decalactone pool, the same differential expression obtained using RNA-seq (Table 2). Quantitative RT-PCR also validated the RNA-seq data for other three up-regulated genes (see below; Additional file 1: Figure S2).

**Figure 4:**
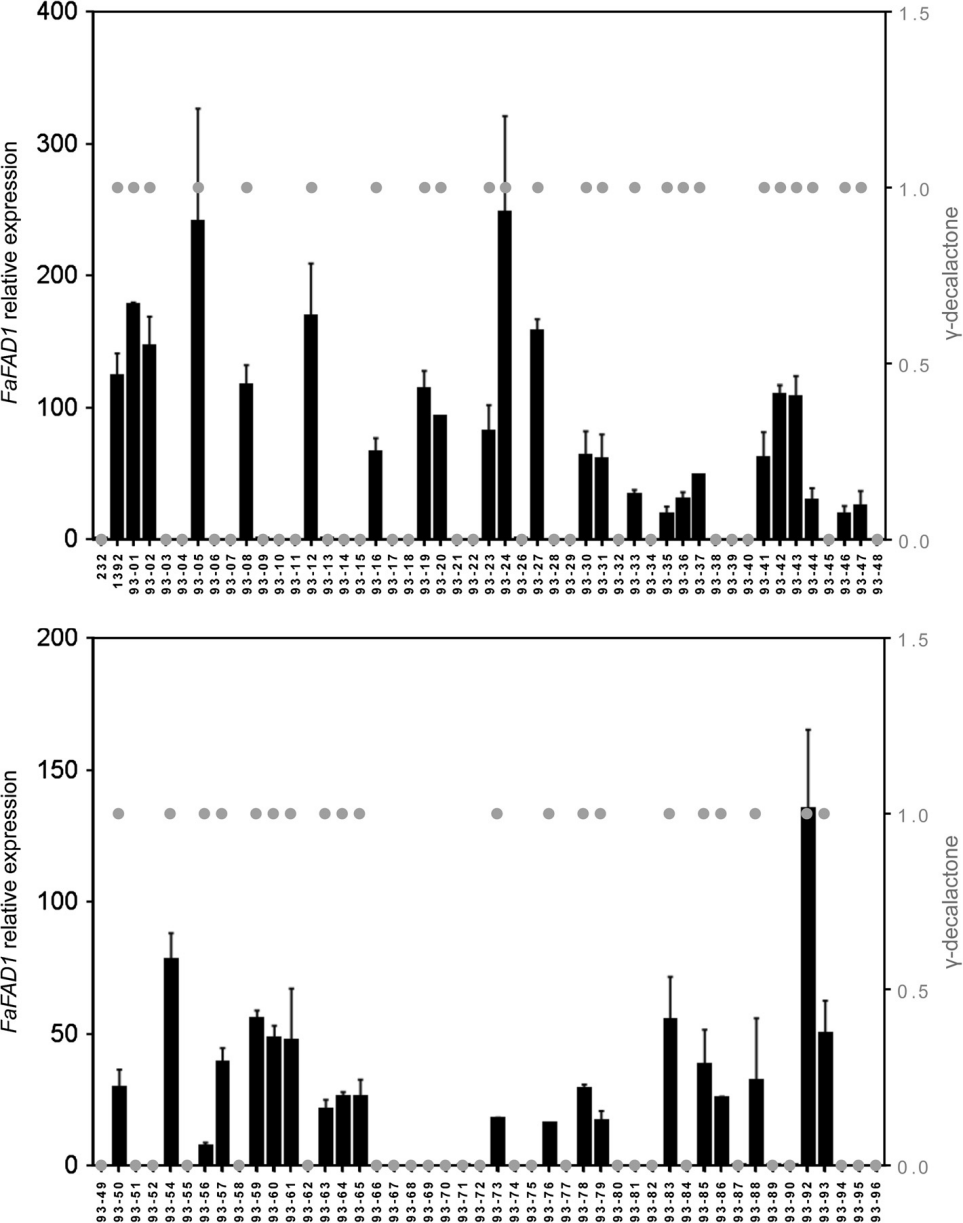
**Comparison of expression of *FaFAD1 *in the 232 × 1392 mapping population by real time qRT-PCR with γ-decalactone content in fruits.** FaFAD1 expression (black bars) is expressed relative to the average expression in the population and it is indicated on the Y-axis to the left. The production of γ-decalactone scored as presence/absence (gray dots) is indicated on the Y-axis to the right.

We next examined the expression level of the gene by qRT-PCR in the complete ‘232’ × ‘1392’ population composed of 93 siblings and the two parental lines. 51 lines (~54%) showed no detectable expression of *FaFAD1*, with threshold cycles (Ct) similar (± 2) to the no-template control and relative expression levels ranging from 0.01 to 0.58 (Figure 4). The rest of the lines showed different levels of expression that ranged from 7 to 247 times the average expression in the population (Figure 4). Furthermore, co-segregation between high/no *FaFAD1* transcription and γ-decalactone content was observed (Figure 4; Additional file 1: Figure S4A). Strikingly, primers used for qRT-PCR of *FaFAD1* failed to amplify in genomic DNA from each line producing fruits without γ-decalactone (Additional file 1: Figure S3A).

**Figure 5:**
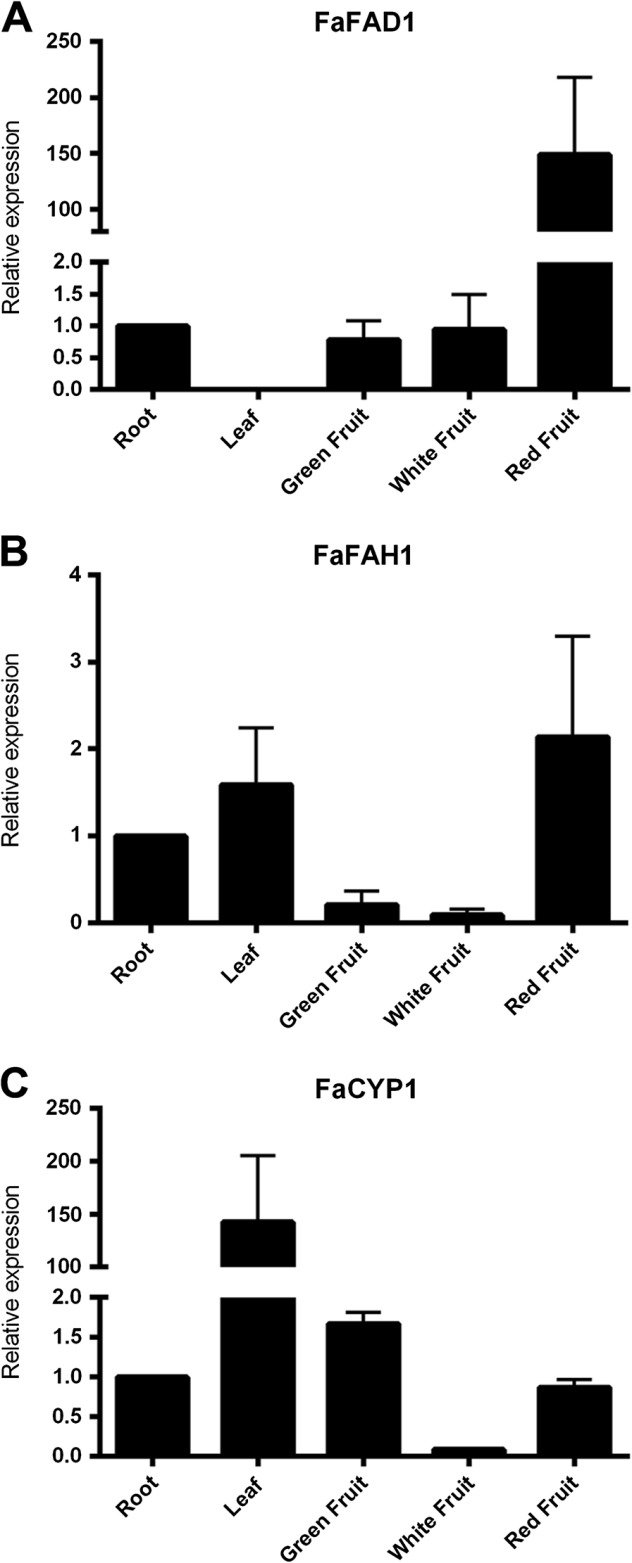
**Expression profiles of *candidate genes *in different tissues and during fruit ripening determined by real-time qRT-PCR analysis. (A)** Expression of *FaFAD1*, **(B)** expression of *FaFAH1* and **(C)** expression of *FaCYP1*. Error bars indicate standard deviations from three biological replicates. Expression levels are expressed as a ratio relative to the root tissue.

Several *FAD2* genes show a seed specific expression, having a role in seed storage fatty acids, while others have an ubiquitous expression, being then involved in the general biosynthesis of membrane fatty acids [36]. This correlation between expression and function of other desaturases prompted us to analyze the expression pattern of *FaFAD1* in order to determine whether it is correlated with the induction of γ-decalactone production during the last stages of fruit ripening. The expression in different tissues and during fruit ripening was analyzed by qRT-PCR in the commonly cultivated Chandler cultivar. In this cultivar, the expression level of *FaFAD1* in red fruits was similar to that of line ‘1392’ (Additional file 1: Figure S3B, C), consistent with both genotypes producing γ-decalactone in ripe fruits. In contrast, expression of *FaFAD1* was not detected in ‘232’ and ‘Camarosa’ by qRT-PCR, indicative of both cultivars not producing γ-decalactone. As shown in Figure 5A, *FaFAD1* increased its expression ~150-fold between white and red fruit, consistent with the biosynthesis of γ-decalactone during the late stages of fruit ripening. Supporting a specific role of *FaFAD1* in ripe fruits, no expression was detected in leaves, and very low expression of *FaFAD1* was detected in roots, green and white fruits.

### Collocation of QTL for γ-decalactone content and eQTL for candidate genes

Based in their predicted function, we selected two additional candidate genes for further analyses within the top 25 highly up-regulated genes (Table 2). The fourth transcript in the list, gene17831-v1.0-hybrid, showed 17-fold (4.1 log2_fold_change) higher expression in the high-γ-decalactone pool (Table 2; Additional file 3). The predicted protein sequence has high similarity to the cytochrome P450, family 81, and contains the p450 superfamily conserved domain (E-value 8.37e-96) and the PLN02183 5-hydroxylase multidomain (E-value 1.22e-70). The gene02395-v1.0-hybrid, at the 23rd position in Table 2, showed a 5-fold up-regulation and encodes for a predicted protein with high similarity to the cytochrome P450, family 79, subfamily A and thus, also contains the p450 superfamily conserved domain (E-value 3.60e-40) and several hydroxylase domains such as PLN03018 (E-value 6.14e-125). The differential gene expression observed for these two transcripts was validated by qRT-PCR, obtaining a 14.8 and 6.5 fold-change in their expression between the pools for gene17831 and gene02395, respectively (Additional file 1: Figure S2). Since these two genes encode for protein sequences with high homology to CYP hydroxylases, we further investigated their possible association to γ-decalactone biosynthesis by analyzing their expression in parental and all progeny lines of the ‘232’ × ‘1392’ mapping population (Additional file 1: Figure S4). A significant level of correlation between the transcript level of the *F. × ananassa* gene corresponding to gene17831-v1.0-hybrid (hereafter referred to as *FaFAH1*) and γ-decalactone content was observed (Pearson correlation = 0.45). On the contrary, no association between the transcript level of gene02395-v1.0-hybrid (hereafter referred to as *FaCYP1*) and γ-decalactone content was observed. Furthermore, expression profiling in different tissues by qRT-PCR showed that *FaFAH1* is expressed in leaf and ripe fruits while *FaCYP1* was highly expressed in leaf and to a much lesser extend in green fruit (Figure 5B, C). These results are consistent with *FaFAH1* but not *FaCYP1* having a possible role in γ-decalactone accumulation in ripening strawberries.

**Figure 6:**
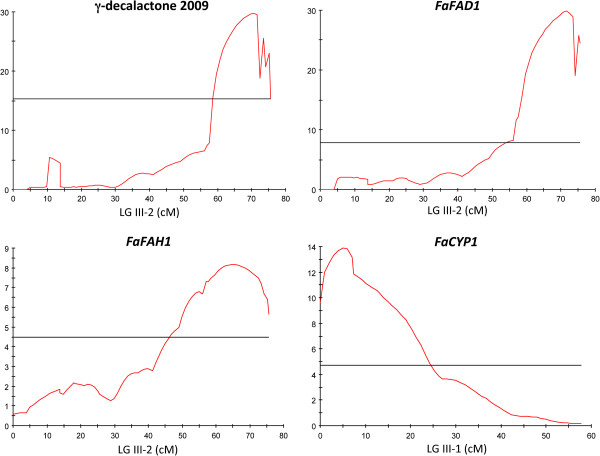
**LOD profiles for γ-decalactone content and *FaFAD1*, *FaFAH1 *and *FaCYP1 *expression (shown in red) on linkage group III-2 for the former three and on LG III-1 for *FaCYP1 *(position is indicated in cM).** Horizontal line marks the significant threshold for each QTL.

Transcript expression levels measured for genes in a mapping population allow them to be treated as traits for gene expression QTL (eQTL) analysis. The locations of eQTL that regulate gene expression can be correlated with those of QTL for traditional phenotypic traits and so provide additional clues as to the genetic basis of quantitative genetic variation [37]. The complete correlation observed between high or no *FaFAD1* expression and γ-decalactone content (Figure 4; Additional file 1: Figure S4A) indicates that an eQTL controlling the expression of *FaFAD1* collocates with both the position of the gene and the phenotypic trait. In order to evaluate this prediction and to test whether the differential expression of *FaFAH1* or *FaCYP1* could be correlated with the position of the locus controlling γ-decalactone content, we next used the qRT-PCR quantitative data for eQTL analysis for the three genes. As a positive control we re-analyzed the QTL for γ-decalactone content [6]. Interestingly, QTL analysis performed with both the non-parametric Kruskal-Wallis test and interval mapping using the integrated map of [6] resulted in the identification of the same eQTL for both *FaFAD1* and *FaFAH1* expression (Figure 6; Additional file 1: Table S5). The eQTL for *FaFAD1* expression, as expected, collocated with both the position of the QTL controlling γ-decalactone content and the position of the gene *FaFAD1* at the bottom of LG III-2 and accounted for 90% of the variation (Additional file 1: Table S5). Interestingly, an eQTL controlling 55% of the variation in the expression of *FaFAH1* was detected also in LG III-2, at the exact same position as where *FaFAD1* (and the *locus* controlling γ-decalactone content) is mapped. The position of *FaFAH1* is predicted to be in one LG of HG I based in the *F. vesca* genome sequence (Table 2), implying that the eQTL at LG III, which most likely is the gene *FaFAD1*, is regulating the expression of *FaFAH1*. When we analyzed the expression of *FaCYP1*, an eQTL was detected at the top of a different LG belonging to the same homoeology group III (Figure 6; Additional file 1: Table S5). The position of the eQTL for FaCYP1 matches the position of the gene based in the *F. vesca* genome sequence (Table 2). This data is consistent with the lack of association previously found between the transcript level of *FaCYP1* and γ-decalactone content.

## Discussion

Two bulked pools of segregants representing the phenotypic extremes within a relatively large population displaying wide variation for a given trait would only differ at the *locus* controlling the trait. Although bulk segregant analysis (BSA) has generally being used to tag genes controlling Mendelian traits, the method can also be used to identify major QTL [38]. The applicability of BSA to RNA-seq was recently demonstrated by mapping the maize mutant gene *gl3*[39]. Here we report the combination of BSA and RNA-seq as a powerful and valid approach for quantifying differential transcript expression and for cost-efficient identification of genes underlying γ-decalactone variation in cultivated strawberry. Once we fine mapped the *locus* to the bottom of chromosome 3, the assumptions made for candidate genes were that (1) the genes must show low or no expression in fruits without γ-decalactone while in fruits producing this VOC had to be high and (2) the gene must encode for an enzyme involved in the biosynthesis of this volatile, based on the proposed pathways, or should encode for a regulatory protein. Out of the 33,458 analyzed transcripts, only gene24414 fulfilled both requirements. This gene encodes for a protein, FaFAD1, with extensive similarity to delta-twelve fatty acid desaturases, enzymes that catalyze the regioselective introduction of a double bond at the Δ12 position during lipid biosynthesis [34]. Therefore, the activity of this protein could supply fatty acid precursors for lactone biosynthesis. The sequence alignment of FaFAD1 with other desaturases revealed the presence of three conserved histidine boxes reported to be essential for the catalysis, and proposed to be the ligands for the iron atoms involved in the formation of the di-iron-oxygen complex. Interestingly, the deduced FaFAD1 protein is shorter than the rest of FAD proteins and neither the dilysine nor the aromatic amino acid-enriched retrieval signal (-YKNKF) are present at the C-terminus of FaFAD1 (Figure 2). One of these motifs is necessary for maintaining localization of the enzymes in the endoplasmic reticulum (ER) [40]. However, a PSORT algorithm (http://wolfpsort.org) predicts that FaFAD1 is targeted to the ER with a certainty of 8.0, consistent with the six transmembrane domains predicted for FaFAD1.

In addition to a desaturase activity, a number of FAD2 variants are known to possess diversified functionalities, catalyzing hydroxylations, epoxidations, or the formation of acetylenic and conjugated double bonds [35,41,42]. Some other FAD2 enzymes have bifunctional hydroxylase/desaturase or even tri-functional activities [43,44]. A close homologue to FaFAD1 in peach, PpFAD1B-6, has been proposed to be involved in lactone production in fruits [14]. This enzyme inserts a double bond between carbon 12 and 13 of monounsaturated oleic acid to generate polyunsaturated linoleic acid, but do not have any detectable hydroxylase activity. However, FaFAD1 is phylogenetically located in a different clade and more closely related to the castor bean hydroxylase RcFAH12 [35]. Seven amino acid residues that differ between oleate desaturases and hydroxylases have been identified and the substitutions of alanine 148 and methionine 324 of the Arabidopsis AtFAD2 by isoleucines, as found in RcFAH12 or *Lesquerella fendleri* hydroxylase/desaturase (LfFAH12), caused a substantial shift in catalytic activity [45,46]. Interestingly, these two isoleucines are conserved between FaFAD1 and hydroxylases, suggesting that the strawberry gene could encode for a bifunctional enzyme (Figure 2).

The expression profiling of *FaFAD1* in different tissues showed that the gene is highly expressed and specific of red fruit of lines with high γ-decalactone content. Therefore, the expression is highly correlated with γ-decalactone biosynthesis, which occurs at the late stages of fruit ripening [4]. In addition, the correlation of *FaFAD1* expression with γ-decalactone content in the mapping population, the coincident map position between γ-decalactone and *FaFAD1* and the predicted enzymatic activity of FaFAD1 protein indicate that this gene is responsible for the natural variation of this VOC in strawberry. Furthermore, it can be stated that the absence or extremely low levels of γ-decalactone in fruits of half of the population lines is a consequence of the absence or extremely low levels of *FaFAD1* expression in these lines. The same *FaFAD1* allele was detected in both bulked pools either using the reference genome to map the reads or after *de novo* assembly. The differential expression of *FaFAD1* observed between both pools was alike using both methods (Additional file 1: Table S6) and was also validated by qRT-PCR (Additional file 1: Figure S2; Table S6). However, when the progeny lines were analyzed independently, *FaFAD1* expression was not detected by qRT-PCR in fruits without γ-decalactone. Furthermore, different *FaFAD1* primer pairs failed to amplify in genomic DNA of these lines (Additional file 1: Figure S3; see also companion manuscript), suggesting that the *FaFAD1* gene may not be present in their genome. Taking these results together, the most plausible explanation is that the no γ-decalactone pools had some contamination during processing with some fruits containing the volatile.

Two other candidate genes were studied on their potential contribution to γ-decalactone production based on their increased expression in the high γ-decalactone pool and the annotated enzymatic activity. While *FaCYP1* was not associated to γ-decalactone content, the gene *FaFAH1* was up-regulated during fruit ripening. Our eQTL analysis of *FaFAD1* and *FaFAH1* indicate that both are associated with γ-decalactone. While the association of *FaFAD1* expression with γ-decalactone is complete, *FaFAH1* only shows a high association with γ-decalactone. When one eQTL maps in the same genetic location as the gene whose transcript is being measured, as it is the case for *FaFAD1*, is generally caused by cis-acting regulatory polymorphisms in the gene (cis-eQTL). Most probably through a polymorphism in the promoter region, which in turn gives rise to differential expression. In contrast, eQTL that do not map to the location of the gene being assayed, such as for *FaFAH1,* most likely represent trans-acting regulators (trans-eQTL) that may control the expression of a number of genes elsewhere in the genome [37]. Based in the predicted function of FaFAD1 and FaFAH1, we propose that the pathway for γ-decalactone biosynthesis in fruits proceeds through hydration of unsaturated fatty acids. In this proposed model, the enzyme FaFAD1 would catalyze the conversion of oleic acid (18:1) to linoleic acid (18:2) by the introduction of a double bond at the Δ12 position, as performed by other FAD2 enzymes. Additionally, FaFAD1 may possess hydroxylase activity, catalyzing the hydroxylation of oleic acid to ricinoleic acid. The fact that an eQTL for *FaFAH1* expression was detected at the position where *FaFAD1* maps suggests that FaFAD1, or most likely the product of FaFAD1 activity (i.e. linoleic acid), up-regulates the expression of *FaFAH1*, which may encode for the enzyme catalyzing the next reaction in the biosynthetic pathway. This reaction most probably is a hydroxylation although some CYP related enzymes have been shown to have epoxidase activity [47]. Ricinoleic acid derivative is then shortened by four β-oxidation cycles to form the corresponding 4-hydroxy acid. The last step in γ-decalactone biosynthesis involves the cyclation of the molecule either by an enzyme with alcohol acyl-transferase activity or by spontaneous lactonisation under acid conditions [48].

## Conclusions

Understanding the basis of volatile organic compound (VOC) biosynthesis and regulation is of utmost importance for the genetic improvement of fruit flavor. This study provides genetic and molecular data on how the content of γ-decalactone is naturally controlled in strawberry and highlights enzymatic activities necessary for the formation of this VOC in fruits. γ-decalactone has been shown to be a sensory important VOC for strawberry flavor [17,49]. However, other important functions of volatiles are to defend plants against pathogens, to attract pollinators, seed dispersers, and other beneficial animals and microorganisms, and to serve as signals in plant–plant interaction [50]. GO enrichment analysis for the genes up-regulated in fruits without γ-decalactone detected a significant enrichment in GO categories related to response to pathogens. One plausible explanation is that this lactone could have anti-pathogen activity and, in its absence, up-regulation of other mechanisms of biotic stress responses would compensate the lack of this VOC. In this context, γ-decalactone has been shown to be toxic to yeast and bacteria through its capacity for permeabilizing membranes [48]. These data suggest that this VOC might have a function in this process, a possibility that deserves further investigation.

### Availability of supporting data

The data sets supporting the results of this article are included within the article (and its additional files) and raw RNA-seq reads available in the European Nucleotide Archive (ENA) repository under accession PRJEB5430 (http://www.ebi.ac.uk/ena/data/view/PRJEB5430).

## Competing interests

The authors would like to declare that they have no financial or non-financial competing interests in the publication of this manuscript.

## Authors’ contributions

JS-S carried out the RNA-seq analysis and bioinformatics studies and participated to draft the manuscript. EC-R participated in qRT-PCR analysis and gene cloning. VV participated in the sequence alignment and helped to draft the manuscript. MB participated in the design of the study and helped to draft the manuscript. IA conceived of the study, carried out the genetic analyses and RNA isolation, participated in its design and coordination and drafted the manuscript. All authors read and approved the final manuscript.

## Supplementary Material

Additional file 1: Figure S1 Distribution of differentially expressed genes in major functional terms (GO terms) for categories Biological Process, Molecular Function and Cellular Component. **Figure S2.** Validation of RNA-seq data by qRT-PCR. **Figure S3.** Polymorphism of *FaFAD1* and expression analysis in ripe fruits of different accessions of *Fragaria × ananassa.***Figure S4.** Expression profiles of candidate genes in the ‘232’ x ‘1392’ mapping population by real time qRT-PCR in comparison to γ-decalactone content in fruits. **Table S1.** Primers used in qRT-PCR. **Table S2.** Summary of read alignments for the three high-γ-decalactone (H γ-DEC) and three not producing γ-decalactone (No γ-DEC) biological replicates. **Table S3.** GO enrichment analysis by mean of Fisher’s exact test with the sets of up-regulated genes/locus (highly expressed in high-γ-decalactone pool) in comparison to general model of *Fragaria vesca.***Table S4.** GO enrichment analysis by mean of Fisher’s exact test with the sets of down-regulated genes/locus (higher expression in the No-γ-decalactone pool) in comparison to general model of *Fragaria vesca.***Table S5.** QTL detected in the ‘232’ × ‘1392’ strawberry population controlling the content of γ-decalactone and eQTL controlling the expression of *FaFAD1*, *FaFAH1* and *FaCYP1* based on Kruskal-Wallis (K-W) and interval mapping (IM). **Table S6.** Expression of gene24414-v1.0-hybrid in each of the biological replicates by three different approaches. Click here for file

Additional file 2 List of the SSR, SNP, AFLP and gene markers mapped in the HG III along with DArT sequence, SNP position and their genotypes in the 232 × 1392 population. Click here for file

Additional file 3 RNA-seq expression values for differentially expressed genes and for all analyzed genes. Click here for file
