# Supplementary material for: Deciphering gamma-decalactone biosynthesis in strawberry fruit using a combination of genetic mapping, RNA-Seq and eQTL analyses
Source: BMC Genomics. 2014 Apr 17;15:218. doi: 10.1186/1471-2164-15-218 (PMC4023230; doi:10.1186/1471-2164-15-218)
Supplement: Additional file 1: Figure S1 — Distribution of differentially expressed genes in major functional terms (GO terms) for categories Biological Process, Molecular Function and Cellular Component. Figure S2. Validation of RNA-seq data by qRT-PCR. Figure S3. Polymorphism of FaFAD1 and expression analysis in ripe fruits of different accessions of Fragaria × ananassa.Figure S4. Expression profiles of candidate genes in the ‘232’ x ‘1392’ mapping population by real time qRT-PCR in comparison to γ-decalactone content in fruits. Table S1. Primers used in qRT-PCR. Table S2. Summary of read alignments for the three high-γ-decalactone (H γ-DEC) and three not producing γ-decalactone (No γ-DEC) biological replicates. Table S3. GO enrichment analysis by mean of Fisher’s exact test with the sets of up-regulated genes/locus (highly expressed in high-γ-decalactone pool) in comparison to general model of Fragaria vesca.Table S4. GO enrichment analysis by mean of Fisher’s exact test with the sets of down-regulated genes/locus (higher expression in the No-γ-decalactone pool) in comparison to general model of Fragaria vesca.Table S5. QTL detected in the ‘232’ × ‘1392’ strawberry population controlling the content of γ-decalactone and eQTL controlling the expression of FaFAD1, FaFAH1 and FaCYP1 based on Kruskal-Wallis (K-W) and interval mapping (IM). Table S6. Expression of gene24414-v1.0-hybrid in each of the biological replicates by three different approaches. [file 1471-2164-15-218-S1.pdf]

Additional file 1

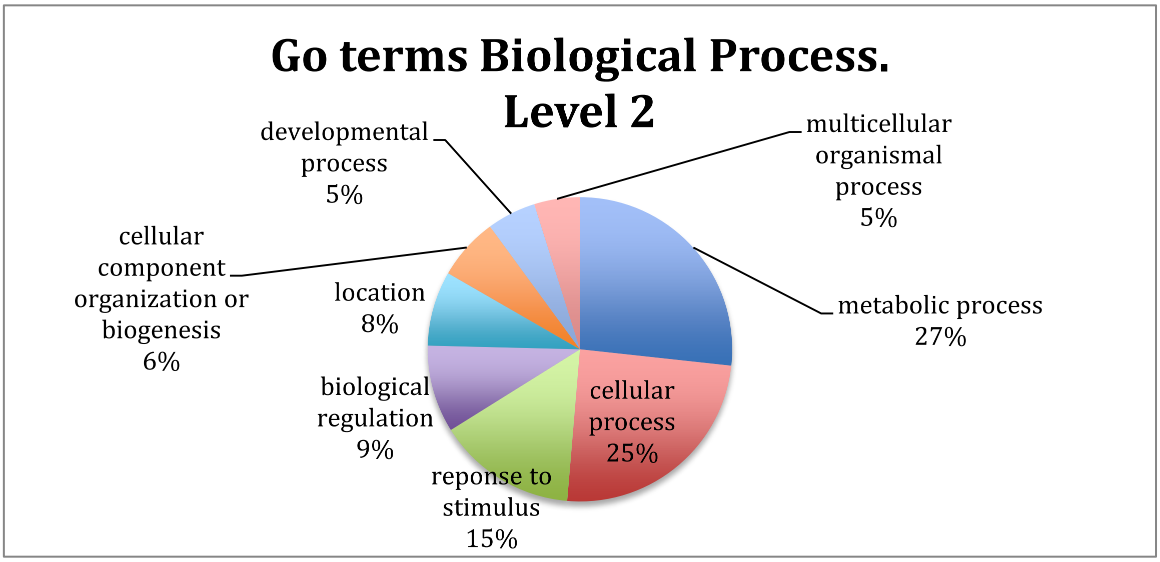

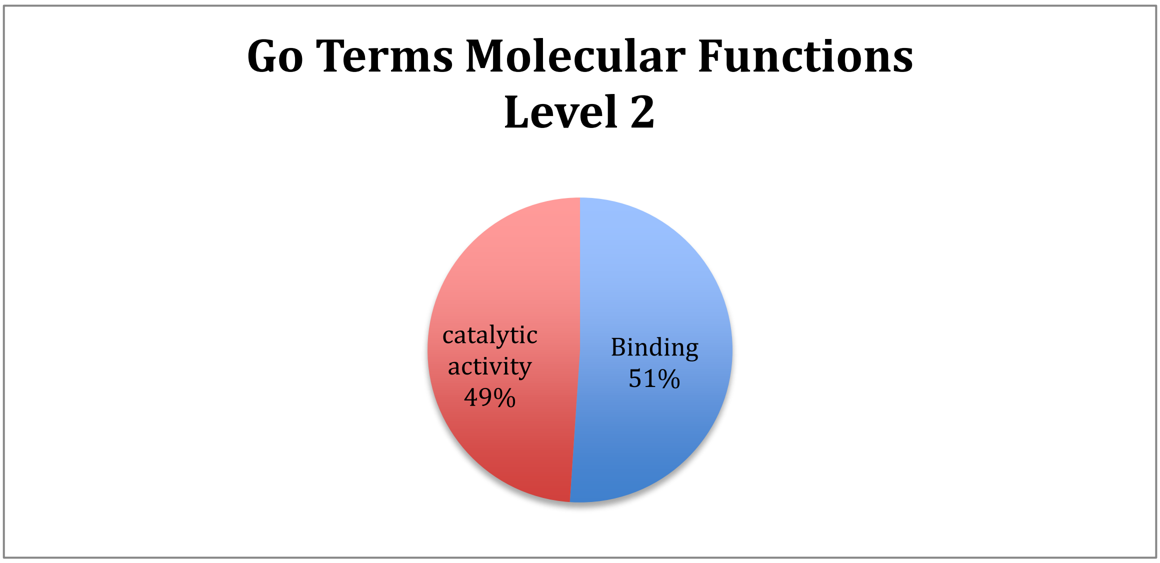

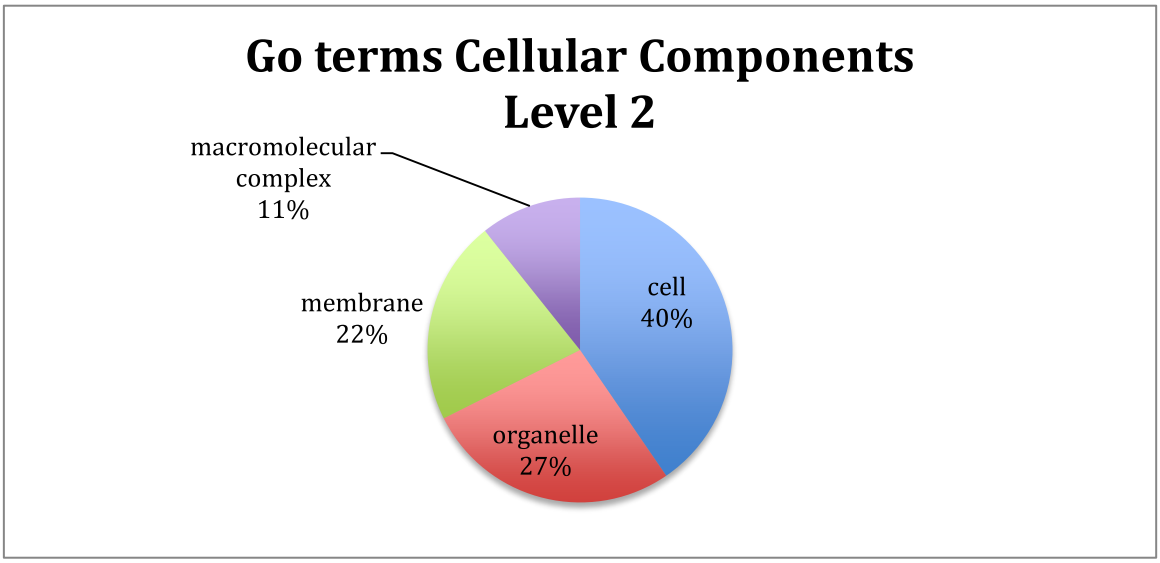

Figure S1 Distribution of differentially expressed genes in major functional terms (GO terms) for categories Biological Process, Molecular Function and Cellular Component

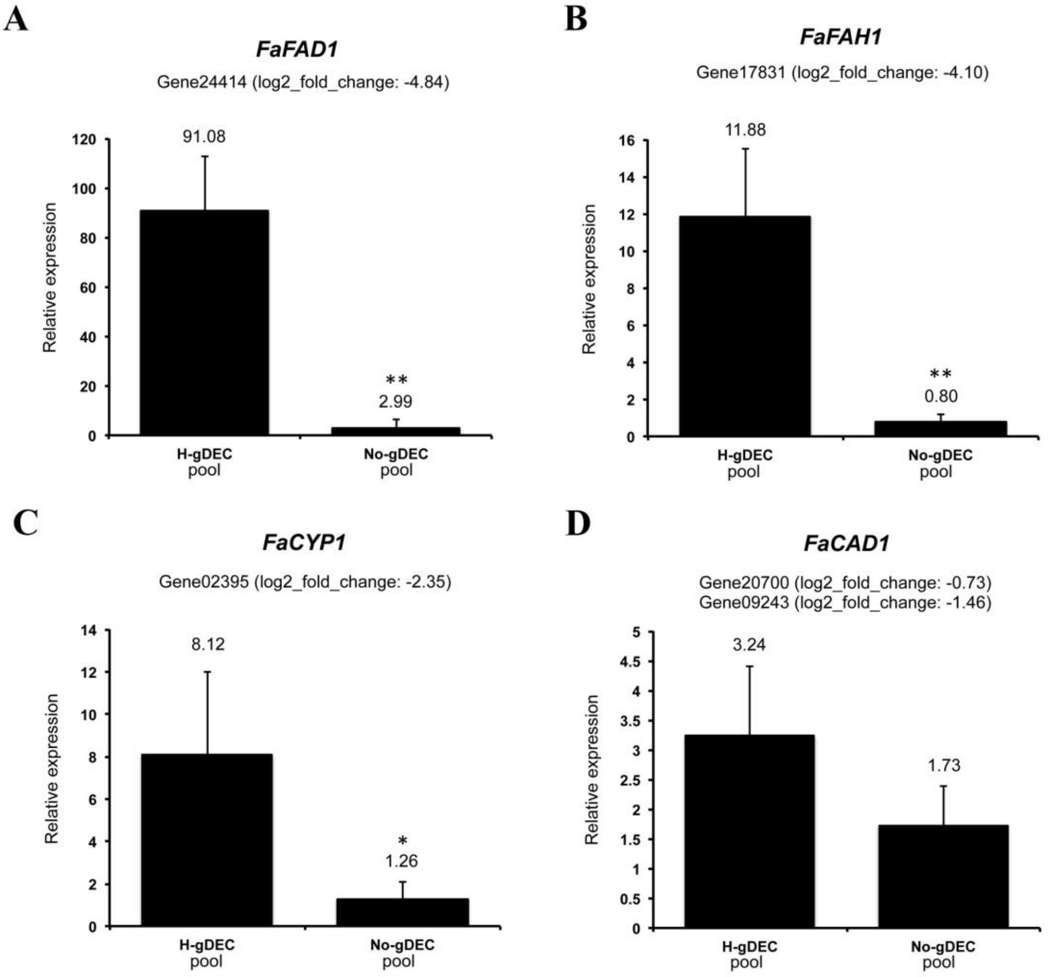

Figure S2 Validation of RNA-seq data by qRT-PCR. The relative expression of *FaFAD1* (A), *FaFAH1* (B), *FaCYP1* (C) and *FaCAD1* was tested by qRT-PCR in the three biological replicates of the two pools used for RNA-seq. Error bars represent standard deviations and asterisks denote significant differences by Student's t test (* $P<0.05$; ** $P<0.01$). For each gene, the fold-change (in log2) obtained in the RNA-seq is depicted on top of the graphs as well as the predicted gene IDs in the *F. vesca* genome.

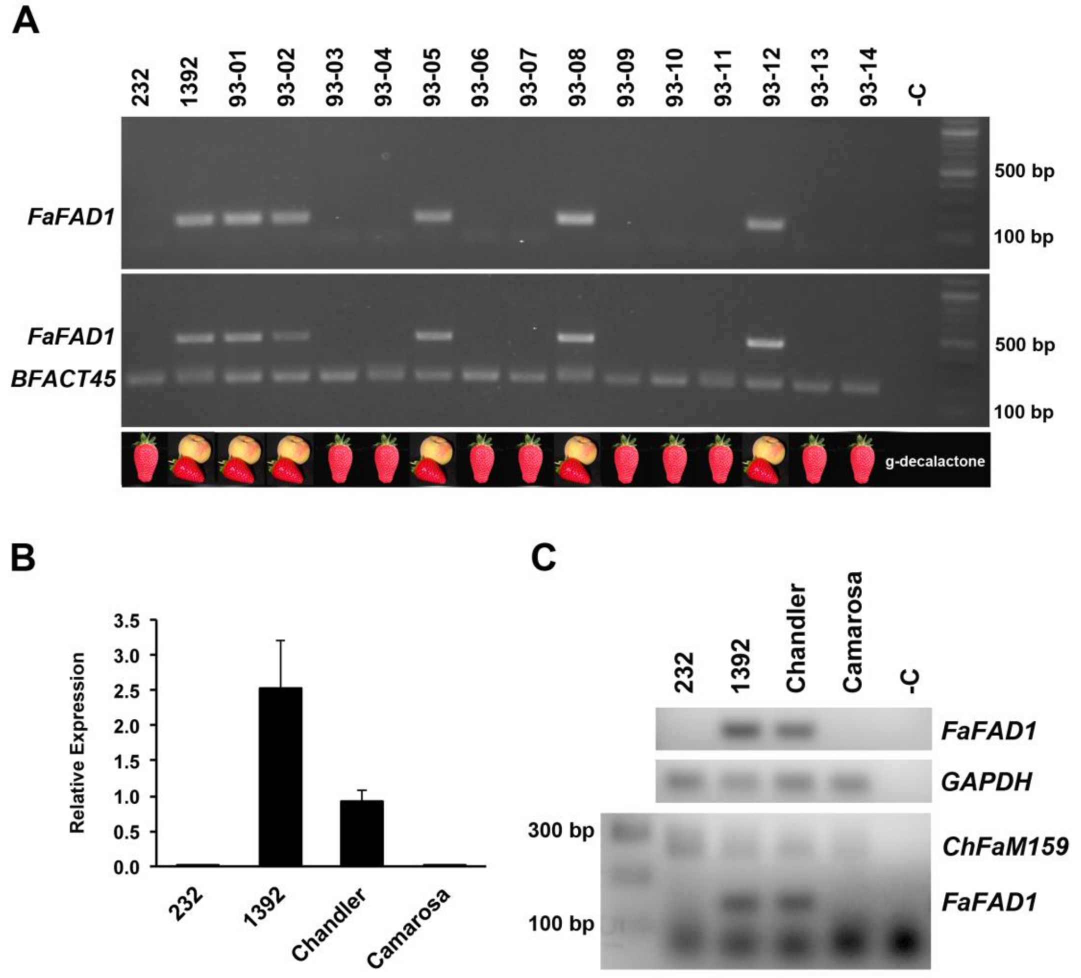

Figure S3 Genomic DNA from parental and 14 progeny lines screened with primers used in the qRT-PCR (**A**, top panel) or with the *FaFAD1* molecular marker developed in the companion paper by Chambers et al. (**A**, lower panel). Although 16 lines are shown in the figure, co-segregation between markers and γ-decalactone content (indicated by a peach below the gels) was observed for all mapping individuals. **B**. Relative expression of *FaFAD1* in ripe fruits of different accessions of *Fragaria × ananassa* determined by qRT-PCR. Expression levels are expressed as a ratio relative to Chandler. **C.** Expression analysis of *FaFAD1* in the same accessions by semi-quantitative RT-PCR (top panel). The number of amplification cycles was 29 and 25 for *FaFAD1* and the constitutive *GADPH* genes, respectively. The lower panel depicts *FaFAD1* amplification on genomic DNA from the same cultivars. SSR marker ChFaM159 (250-260 bp; Zorrilla-Fontanesi Y, Cabeza A, Torres A, Botella M, Valpuesta V, Monfort A, Sánchez-Sevilla J, Amaya I: **Development and bin mapping of strawberry genic-SSRs in diploid *Fragaria* and their transferability across the Rosoideae subfamily**. *Mol Breed* 2011, **27**:137–156) was used as positive control in the PCR.

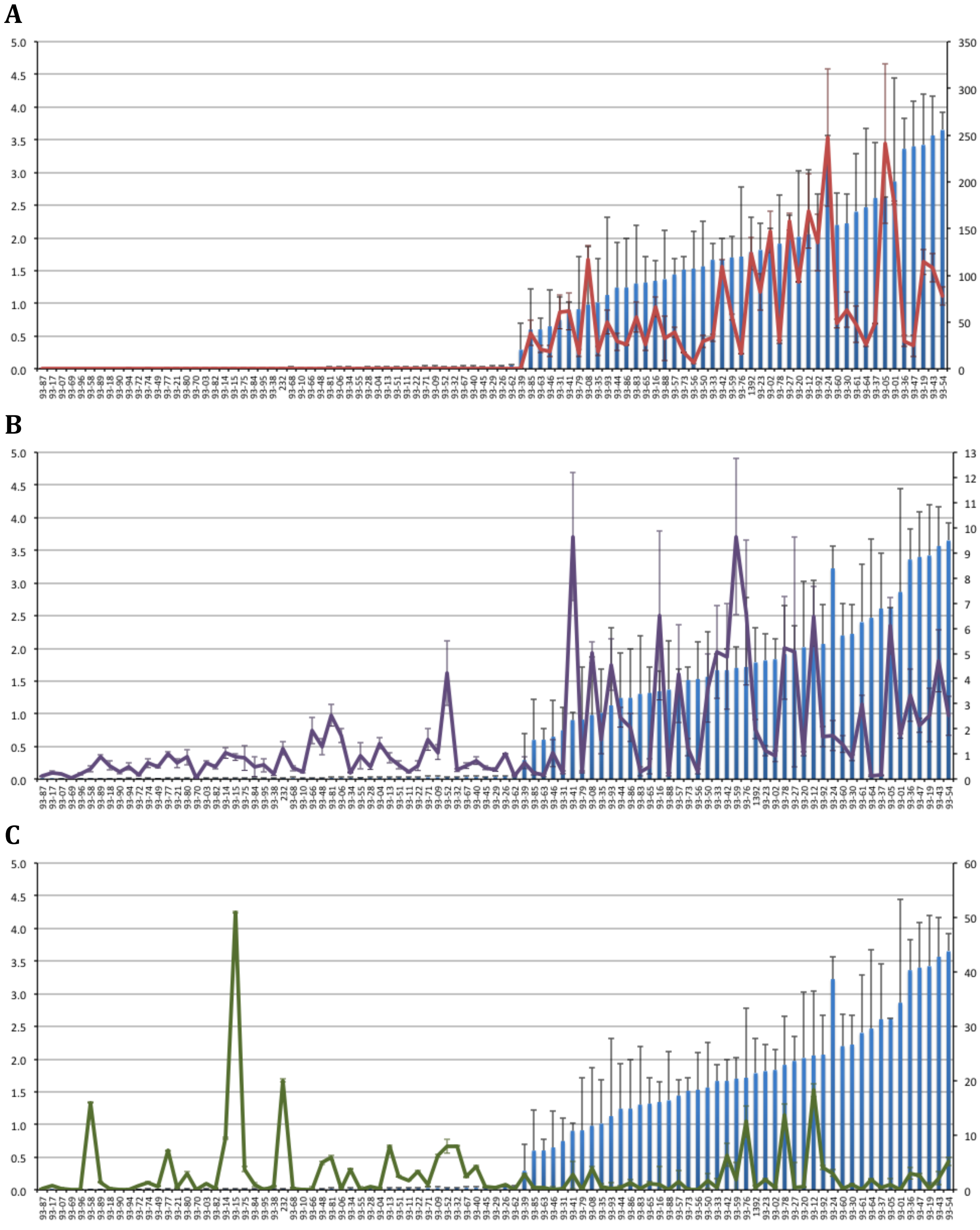

Figure S4 Expression profiles of candidate genes in the ‘232’ x ‘1392’ mapping population by real time qRT-PCR in comparison to γ-decalactone content in fruits. *FaFAD1* (A), *FaFAH1* (B) and *FaCYP1* (C) expression (red, purple and green lines, respectively) is expressed relative to the average expression in the population and it is indicated on the Y-axis to the right. The average content of γ-decalactone for seasons 2007-2009 (blue bars) is indicated on the Y-axis to the left. Pearson correlation coefficients between each gene and γ-decalactone content were 0.73, 0.45 and -0.06 for *FaFAD1*, *FaFAH1* and *FaCYP1*, respectively.

Table S1 Primers used in qRT-PCR.

Gene	Primer	Sequence 5´-3'	Product size (bp)
FaFAD1	Forward	TCTGTACTCTACCGCCTTGC	140
	reverse	TCGTAGTGTGGCAGTGAAGG	
FaFAH1	Forward	CCTTTCATACGGCGGGGGAA	157
	reverse	CGAAGCTCTCTTGTTCCGGTG	
FaCYP1	Forward	ATGCTCTTTGCCAGGCTTCT	132
	reverse	AGCCAAGCGAGGTTTAGCAT	
FaCAD1	Forward	TTCCAGGGCATGAGATTGTTGG	201
	reverse	CATAGGTGGTGCTTCCGTCG	
GAPDH	Forward	TCCATCACTGCCACCCAGAAGACTG	132
	reverse	AGCAGGCAGAACCTTTCCGACAG	

Table S2 Summary of read alignments for the three high-γ-decalactone (H γ-DEC) and three not producing γ-decalactone (No γ-DEC) biological replicates. M, millions.

ID	Total Reads	Clean Reads	Paired in Sequencing		Properly paired	With itself and mate mapped	Singletons	Mate mapped to a different chrom.
			Reads	Pair 1				
		(%)	(%)	Pair 2	(%)		(%)	mapQ>=5
H γ-DEC 1	30.10 M	29.24 M	40.18 M	20.35 M	27.31 M	32.61 M	7.57 M	0.23 M
		97.13%	68.72%	19.83 M	67.96%		18.85%	0.09 M
H γ-DEC 2	36.16 M	35.22 M	48.96 M	24.66 M	33.31 M	39.88 M	9.08 M	0.29 M
		97.38%	69.52%	24.30 M	68.02%		18.55%	0.11 M
H γ-DEC 3	36.61 M	35.40 M	49.16 M	24.92 M	32.42 M	39.76 M	9.39 M	0.30 M
		96.69%	69.43%	24.24 M	65.95%		19.11%	0.11 M
No γ-DEC 1	40.91 M	39.77 M	55.35 M	27.89 M	38.40 M	45.25 M	10.10 M	0.30 M
		97.21%	69.59%	27.46 M	69.37%		18.25%	0.12 M
No γ-DEC 2	35.90 M	34.49 M	48.33 M	24.34 M	33.28 M	39.62 M	8.71 M	0.27 M
		97.44%	70.06%	24.00 M	68.86%		18.03%	0.10 M
No γ-DEC 3	38.33 M	37.45 M	52.14 M	26.25 M	36.00 M	42.75 M	9.39 M	0.29 M
		97.71%	69.62%	25.89 M	69.05%		18.02%	0.11 M

Table S3 GO enrichment analysis by mean of Fisher's exact test with the sets of up-regulated genes/locus (highly expressed in high-γ-decalactone pool) in comparison to general model of *F. vesca*. Category: P: Biological Process, F: Molecular function, C: Cellular component. FDR: The value of the Fisher's test statistic used to compute significance. P-values represent significant differences between the number of genes assigned to the GO category in the reference *F. vesca* background and in the high-γ-decalactone-induced genes. # Test: Number of sequences annotated in the set.

GO-ID	Term	Category	FDR	P-Value	#Test
GO:0055114	oxidation-reduction process	P	2,00E-010	2,64E-014	92
GO:0016491	oxidoreductase activity	F	1,06E-008	2,81E-012	86
GO:0016616	oxidoreductase activity, acting on the CH-OH group of donors, NAD or NADP as acceptor	F	1,42E-006	7,31E-010	24
GO:0016614	oxidoreductase activity, acting on CH-OH group of donors	F	1,42E-006	7,50E-010	25
GO:0042203	toluene catabolic process	P	1,50E-006	1,98E-009	6
GO:0042184	xylene catabolic process	P	1,50E-006	1,98E-009	6
GO:0072491	toluene-containing compound catabolic process	P	1,50E-006	1,98E-009	6
GO:0072490	toluene-containing compound metabolic process	P	1,50E-006	1,98E-009	6
GO:0018970	toluene metabolic process	P	1,50E-006	1,98E-009	6
GO:0018948	xylene metabolic process	P	1,50E-006	1,98E-009	6
GO:0006803	glutathione conjugation reaction	P	2,56E-006	4,06E-009	8
GO:0004364	glutathione transferase activity	F	2,56E-006	4,06E-009	8
GO:0018879	biphenyl metabolic process	P	1,61E-005	2,97E-008	5
GO:0018456	aryl-alcohol dehydrogenase (NAD+) activity	F	1,61E-005	2,97E-008	5
GO:0044455	mitochondrial membrane part	C	1,68E-005	3,32E-008	14
GO:0046029	mannitol dehydrogenase activity	F	1,70E-005	3,82E-008	6
GO:0031320	hexitol dehydrogenase activity	F	1,70E-005	3,82E-008	6
GO:0006950	response to stress	P	2,24E-005	5,32E-008	99
GO:0004033	aldo-keto reductase (NADP) activity	F	3,21E-005	8,06E-008	7
GO:0022626	cytosolic ribosome	C	3,30E-005	8,72E-008	20
GO:0044445	cytosolic part	C	1,99E-004	5,53E-007	21
GO:0044429	mitochondrial part	C	2,65E-004	7,69E-007	19
GO:0022625	cytosolic large ribosomal subunit	C	2,89E-004	8,79E-007	11
GO:0018883	caprolactam metabolic process	P	3,04E-004	1,04E-006	5
GO:0045551	cinnamyl-alcohol dehydrogenase activity	F	3,04E-004	1,04E-006	5
GO:0019384	caprolactam catabolic process	P	3,04E-004	1,04E-006	5
GO:0050896	response to stimulus	P	4,52E-004	1,65E-006	146
GO:0072340	cellular lactam catabolic process	P	4,52E-004	1,67E-006	5
GO:0009607	response to biotic stimulus	P	7,26E-004	2,78E-006	42
GO:0070401	NADP+ binding	F	7,26E-004	2,88E-006	4
GO:0044391	ribosomal subunit	C	9,24E-004	4,02E-006	15
GO:0008152	metabolic process	P	9,24E-004	4,02E-006	256
GO:0005730	nucleolus	C	9,24E-004	4,02E-006	25
GO:0005746	mitochondrial respiratory chain	C	1,36E-003	6,10E-006	9
GO:0031966	mitochondrial membrane	C	1,45E-003	6,68E-006	15
GO:0009409	response to cold	P	1,45E-003	6,90E-006	24
GO:0016758	transferase activity, transferring hexosyl groups	F	1,72E-003	8,42E-006	24
GO:0043605	cellular amide catabolic process	P	2,05E-003	1,04E-005	5
GO:0016846	carbon-sulfur lyase activity	F	2,05E-003	1,06E-005	8
GO:0003735	structural constituent of ribosome	F	2,15E-003	1,14E-005	20
GO:0042221	response to chemical stimulus	P	2,17E-003	1,18E-005	86
GO:0004462	lactoylglutathione lyase activity	F	2,28E-003	1,26E-005	6

GO:0072341	modified amino acid binding	F	2,44E-003	1,39E-005	5
GO:0005740	mitochondrial envelope	C	2,66E-003	1,55E-005	15
GO:1900750	oligopeptide binding	F	2,73E-003	1,66E-005	4
GO:0043295	glutathione binding	F	2,73E-003	1,66E-005	4
GO:0015934	large ribosomal subunit	C	3,33E-003	2,06E-005	11
GO:0019748	secondary metabolic process	P	3,51E-003	2,23E-005	27
GO:0006749	glutathione metabolic process	P	3,51E-003	2,35E-005	8
GO:0072338	cellular lactam metabolic process	P	3,51E-003	2,36E-005	5
GO:0005743	mitochondrial inner membrane	C	3,51E-003	2,36E-005	13
GO:0004032	alditol:NADP+ 1-oxidoreductase activity	F	3,74E-003	2,57E-005	4
GO:0009266	response to temperature stimulus	P	3,80E-003	2,66E-005	30
GO:0042816	vitamin B6 metabolic process	P	4,22E-003	3,01E-005	5
GO:0016757	transferase activity, transferring glycosyl groups	F	4,48E-003	3,26E-005	26
GO:0070469	respiratory chain	C	4,70E-003	3,47E-005	9
GO:0050236	pyridoxine:NADP 4-dehydrogenase activity	F	6,51E-003	4,90E-005	3
GO:0035251	UDP-glucosyltransferase activity	F	9,25E-003	7,08E-005	13
GO:0042277	peptide binding	F	1,10E-002	8,60E-005	5
GO:0071824	protein-DNA complex subunit organization	P	1,18E-002	9,63E-005	9
GO:0065004	protein-DNA complex assembly	P	1,18E-002	9,63E-005	9
GO:0043450	alkene biosynthetic process	P	1,18E-002	9,63E-005	9
GO:0044085	cellular component biogenesis	P	1,30E-002	1,09E-004	45
GO:1900674	olefin biosynthetic process	P	1,32E-002	1,11E-004	9
GO:0046527	glucosyltransferase activity	F	1,32E-002	1,14E-004	13
GO:0005198	structural molecule activity	F	1,37E-002	1,20E-004	21
GO:0019866	organelle inner membrane	C	1,43E-002	1,26E-004	14
GO:0042178	xenobiotic catabolic process	P	1,44E-002	1,29E-004	6
GO:0008106	alcohol dehydrogenase (NADP+) activity	F	1,45E-002	1,32E-004	4
GO:0009811	stilbene biosynthetic process	P	1,76E-002	1,65E-004	6
GO:0009810	stilbene metabolic process	P	1,76E-002	1,65E-004	6
GO:0046482	para-aminobenzoic acid metabolic process	P	1,80E-002	1,71E-004	5
GO:0042537	benzene-containing compound metabolic process	P	1,92E-002	1,85E-004	6
GO:0043603	cellular amide metabolic process	P	2,09E-002	2,07E-004	6
GO:0006518	peptide metabolic process	P	2,49E-002	2,50E-004	8
GO:0051707	response to other organism	P	2,56E-002	2,67E-004	35
GO:0006970	response to osmotic stress	P	2,56E-002	2,69E-004	25
GO:0000786	nucleosome	C	2,56E-002	2,79E-004	7
GO:0055091	phospholipid homeostasis	P	2,56E-002	2,94E-004	2
GO:0055089	fatty acid homeostasis	P	2,56E-002	2,94E-004	2
GO:0055088	lipid homeostasis	P	2,56E-002	2,94E-004	2
GO:0006294	nucleotide-excision repair, preincision complex assembly	P	2,56E-002	2,94E-004	2
GO:0042171	lysophosphatidic acid acyltransferase activity	F	2,56E-002	2,94E-004	2
GO:0070328	triglyceride homeostasis	P	2,56E-002	2,94E-004	2
GO:0080018	anthocyanin 5-O-glucosyltransferase activity	F	2,56E-002	2,94E-004	2
GO:0080002	UDP-glucose:4-aminobenzoate acylglucosyltransferase activity	F	2,56E-002	2,94E-004	2
GO:0043449	cellular alkene metabolic process	P	3,22E-002	3,74E-004	9
GO:0030639	polyketide biosynthetic process	P	3,23E-002	3,88E-004	6
GO:0030638	polyketide metabolic process	P	3,23E-002	3,88E-004	6
GO:0042181	ketone biosynthetic process	P	3,23E-002	3,88E-004	6
GO:0016765	transferase activity, transferring alkyl or aryl (other than methyl) groups	F	3,30E-002	4,04E-004	8
GO:0016705	oxidoreductase activity, acting on paired donors, with incorporation or reduction of molecular oxygen	F	3,30E-002	4,06E-004	19
GO:0005840	ribosome	C	3,34E-002	4,15E-004	24

GO:1900673	olefin metabolic process	P	3,34E-002	4,19E-004	9
GO:0050662	coenzyme binding	F	3,48E-002	4,41E-004	17
GO:0032993	protein-DNA complex	C	3,52E-002	4,51E-004	7
GO:0009072	aromatic amino acid family metabolic process	P	3,67E-002	4,75E-004	15
GO:0008194	UDP-glycosyltransferase activity	F	3,85E-002	5,07E-004	15
GO:0034976	response to endoplasmic reticulum stress	P	3,85E-002	5,09E-004	11
GO:0016597	amino acid binding	F	3,87E-002	5,16E-004	6
GO:0005829	cytosol	C	3,98E-002	5,36E-004	50
GO:0010033	response to organic substance	P	4,39E-002	5,97E-004	53
GO:0009055	electron carrier activity	F	4,40E-002	6,04E-004	22
GO:0009699	phenylpropanoid biosynthetic process	P	4,51E-002	6,25E-004	14

Table S4 GO enrichment analysis by mean of Fisher's exact test with the sets of down-regulated genes/locus (higher expression in the No-γ-decalactone pool) in comparison to general model of *Fragaria vesca*. Category: P: Biological Process, F: Molecular function, C: Cellular component. FDR: The value of the Fisher's test statistic used to compute significance. P-values represent significant differences between the number of genes assigned to the GO category in the reference *F. vesca* background and in the No-γ-decalactone-induced genes. # Test: Number of sequences annotaded in the set.

GO-ID	Term	Category	FDR	P-Value	#Test
GO:0071944	cell periphery	C	8,20E-008	1,08E-011	59
GO:0005886	plasma membrane	C	4,34E-007	1,15E-010	50
GO:0042625	ATPase activity, coupled to transmembrane movement of ions	F	2,10E-005	8,32E-009	12
GO:0015405	P-P-bond-hydrolysis-driven transmembrane transporter activity	F	3,73E-005	2,14E-008	15
GO:0015399	primary active transmembrane transporter activity	F	3,73E-005	2,46E-008	15
GO:0042626	ATPase activity, coupled to transmembrane movement of substances	F	2,29E-004	2,12E-007	13
GO:0043492	ATPase activity, coupled to movement of substances	F	2,29E-004	2,12E-007	13
GO:0016820	hydrolase activity, acting on acid anhydrides, catalyzing transmembrane movement of substances	F	2,41E-004	2,54E-007	13
GO:0022804	active transmembrane transporter activity	F	3,29E-004	3,91E-007	20
GO:0065008	regulation of biological quality	P	5,75E-004	7,59E-007	25
GO:0015662	ATPase activity, coupled to transmembrane movement of ions, phosphorylative mechanism	F	7,79E-004	1,13E-006	7
GO:0016020	membrane	C	1,69E-003	2,68E-006	73
GO:0009607	response to biotic stimulus	P	2,12E-003	3,82E-006	26
GO:0009653	anatomical structure morphogenesis	P	2,12E-003	4,06E-006	28
GO:0006811	ion transport	P	2,12E-003	4,20E-006	25
GO:0015748	organophosphate ester transport	P	3,02E-003	7,97E-006	5
GO:0015716	organic phosphonate transport	P	3,02E-003	7,97E-006	5
GO:0015605	organophosphate ester transmembrane transporter activity	F	3,02E-003	7,97E-006	5
GO:0015604	organic phosphonate transmembrane transporter activity	F	3,02E-003	7,97E-006	5
GO:0015416	organic phosphonate transmembrane-transporting ATPase activity	F	3,02E-003	7,97E-006	5
GO:0046034	ATP metabolic process	P	4,33E-003	1,20E-005	11
GO:0051707	response to other organism	P	5,26E-003	1,53E-005	24
GO:0022857	transmembrane transporter activity	F	5,53E-003	1,72E-005	24
GO:0022892	substrate-specific transporter activity	F	5,53E-003	1,75E-005	23
GO:0046351	disaccharide biosynthetic process	P	6,39E-003	2,11E-005	5
GO:0010016	shoot morphogenesis	P	7,19E-003	2,47E-005	12
GO:0005215	transporter activity	F	8,80E-003	3,14E-005	27
GO:0022891	substrate-specific transmembrane transporter activity	F	9,00E-003	3,45E-005	21
GO:0051704	multi-organism process	P	9,00E-003	3,55E-005	28
GO:0015075	ion transmembrane transporter activity	F	9,00E-003	3,56E-005	18
GO:0019829	cation-transporting ATPase activity	F	9,08E-003	3,72E-005	7
GO:0048513	organ development	P	9,21E-003	3,89E-005	29
GO:0048731	system development	P	9,47E-003	4,13E-005	29
GO:0006812	cation transport	P	1,02E-002	4,59E-005	19
GO:0007155	cell adhesion	P	1,02E-002	4,82E-005	6
GO:0022610	biological adhesion	P	1,02E-002	4,82E-005	6

GO:0009312	oligosaccharide biosynthetic process	P	1,20E-002	6,34E-005	5
GO:0051179	localization	P	1,20E-002	6,42E-005	48
GO:0032502	developmental process	P	1,20E-002	6,92E-005	46
GO:0030641	regulation of cellular pH	P	1,20E-002	7,10E-005	2
GO:0035427	purine nucleoside transmembrane transport	P	1,20E-002	7,10E-005	2
GO:0051453	regulation of intracellular pH	P	1,20E-002	7,10E-005	2
GO:0015211	purine nucleoside transmembrane transporter activity	F	1,20E-002	7,10E-005	2
GO:0010184	cytokinin transport	P	1,20E-002	7,10E-005	2
GO:0048367	shoot development	P	1,20E-002	7,23E-005	15
GO:0016787	hydrolase activity	F	1,20E-002	7,37E-005	51
GO:0022621	shoot system development	P	1,20E-002	7,68E-005	15
GO:0030838	positive regulation of actin filament polymerization	P	1,20E-002	7,88E-005	6
GO:0045010	actin nucleation	P	1,20E-002	7,88E-005	6
GO:0044262	cellular carbohydrate metabolic process	P	1,20E-002	7,93E-005	22
GO:0031334	positive regulation of protein complex assembly	P	1,28E-002	8,99E-005	6
GO:0051495	positive regulation of cytoskeleton organization	P	1,28E-002	8,99E-005	6
GO:0032273	positive regulation of protein polymerization	P	1,28E-002	8,99E-005	6
GO:0008514	organic anion transmembrane transporter activity	F	1,29E-002	9,21E-005	5
GO:0010638	positive regulation of organelle organization	P	1,30E-002	9,43E-005	7
GO:0006950	response to stress	P	1,30E-002	9,63E-005	49
GO:0010090	trichome morphogenesis	P	1,32E-002	9,91E-005	7
GO:0050896	response to stimulus	P	1,36E-002	1,04E-004	75
GO:0030041	actin filament polymerization	P	1,39E-002	1,09E-004	6
GO:0007275	multicellular organismal development	P	1,39E-002	1,10E-004	43
GO:0051130	positive regulation of cellular component organization	P	1,50E-002	1,21E-004	7
GO:0043225	anion transmembrane-transporting ATPase activity	F	1,51E-002	1,30E-004	5
GO:0009555	pollen development	P	1,51E-002	1,30E-004	9
GO:0030833	regulation of actin filament polymerization	P	1,51E-002	1,31E-004	6
GO:0032501	multicellular organismal process	P	1,51E-002	1,36E-004	45
GO:0030832	regulation of actin filament length	P	1,51E-002	1,39E-004	6
GO:0032970	regulation of actin filament-based process	P	1,51E-002	1,39E-004	6
GO:0032956	regulation of actin cytoskeleton organization	P	1,51E-002	1,39E-004	6
GO:0008064	regulation of actin polymerization or depolymerization	P	1,51E-002	1,39E-004	6
GO:0006810	transport	P	1,51E-002	1,40E-004	45
GO:0043254	regulation of protein complex assembly	P	1,54E-002	1,47E-004	6
GO:0032271	regulation of protein polymerization	P	1,54E-002	1,47E-004	6
GO:0044248	cellular catabolic process	P	1,54E-002	1,48E-004	30
GO:0010026	trichome differentiation	P	1,63E-002	1,60E-004	7
GO:0016051	carbohydrate biosynthetic process	P	1,84E-002	1,85E-004	18
GO:0051493	regulation of cytoskeleton organization	P	1,84E-002	1,85E-004	6
GO:0042545	cell wall modification	P	1,89E-002	1,93E-004	9
GO:0005986	sucrose biosynthetic process	P	1,89E-002	2,02E-004	3
GO:0000084	S phase of mitotic cell cycle	P	1,89E-002	2,12E-004	2
GO:0006059	hexitol metabolic process	P	1,89E-002	2,12E-004	2
GO:0051320	S phase	P	1,89E-002	2,12E-004	2
GO:0019594	mannitol metabolic process	P	1,89E-002	2,12E-004	2

GO:0019593	mannitol biosynthetic process	P	1,89E-002	2,12E-004	2
GO:0019406	hexitol biosynthetic process	P	1,89E-002	2,12E-004	2
GO:0019401	alditol biosynthetic process	P	1,89E-002	2,12E-004	2
GO:0051234	establishment of localization	P	1,98E-002	2,25E-004	45
GO:0008154	actin polymerization or depolymerization	P	2,00E-002	2,30E-004	6
GO:0006200	ATP catabolic process	P	2,02E-002	2,35E-004	8
GO:0071704	organic substance metabolic process	P	2,26E-002	2,66E-004	30
GO:0005985	sucrose metabolic process	P	2,70E-002	3,23E-004	11
GO:0042623	ATPase activity, coupled	F	2,70E-002	3,24E-004	13
GO:0044087	regulation of cellular component biogenesis	P	2,84E-002	3,46E-004	6
GO:0006073	cellular glucan metabolic process	P	2,84E-002	3,48E-004	15
GO:0009888	tissue development	P	3,13E-002	3,88E-004	17
GO:0015711	organic anion transport	P	3,26E-002	4,09E-004	5
GO:0015860	purine nucleoside transport	P	3,29E-002	4,21E-004	2
GO:0047274	galactinol-sucrose galactosyltransferase activity	F	3,29E-002	4,21E-004	2
GO:0070588	calcium ion transmembrane transport	P	3,40E-002	4,41E-004	3
GO:0003824	catalytic activity	F	3,40E-002	4,44E-004	108
GO:0006754	ATP biosynthetic process	P	3,47E-002	4,63E-004	5
GO:0005975	carbohydrate metabolic process	P	3,47E-002	4,63E-004	31
GO:0009642	response to light intensity	P	3,60E-002	4,85E-004	8
GO:0031224	intrinsic to membrane	C	3,78E-002	5,14E-004	28
GO:0019400	alditol metabolic process	P	3,79E-002	5,20E-004	3
GO:0051258	protein polymerization	P	3,80E-002	5,27E-004	6
GO:0048878	chemical homeostasis	P	3,80E-002	5,32E-004	8
GO:0044459	plasma membrane part	C	4,12E-002	5,82E-004	8
GO:1901135	carbohydrate derivative metabolic process	P	4,17E-002	5,94E-004	27
GO:0009206	purine ribonucleoside triphosphate biosynthetic process	P	4,24E-002	6,21E-004	5
GO:0009201	ribonucleoside triphosphate biosynthetic process	P	4,24E-002	6,21E-004	5
GO:0009145	purine nucleoside triphosphate biosynthetic process	P	4,24E-002	6,21E-004	5
GO:0009628	response to abiotic stimulus	P	4,24E-002	6,30E-004	34
GO:0007015	actin filament organization	P	4,24E-002	6,55E-004	6
GO:0009142	nucleoside triphosphate biosynthetic process	P	4,24E-002	6,57E-004	5
GO:0046700	heterocycle catabolic process	P	4,24E-002	6,59E-004	13
GO:0000904	cell morphogenesis involved in differentiation	P	4,24E-002	6,66E-004	9
GO:0045087	innate immune response	P	4,24E-002	6,70E-004	13
GO:0044270	cellular nitrogen compound catabolic process	P	4,24E-002	6,70E-004	13
GO:0045543	gibberellin 2-beta-dioxygenase activity	F	4,24E-002	6,98E-004	2
GO:0030894	replisome	C	4,24E-002	6,98E-004	2
GO:0043601	nuclear replisome	C	4,24E-002	6,98E-004	2
GO:0043596	nuclear replication fork	C	4,24E-002	6,98E-004	2
GO:0046173	polyol biosynthetic process	P	4,24E-002	6,98E-004	2
GO:0044264	cellular polysaccharide metabolic process	P	4,24E-002	6,98E-004	16
GO:0007010	cytoskeleton organization	P	4,24E-002	7,00E-004	11
GO:0009205	purine ribonucleoside triphosphate metabolic process	P	4,25E-002	7,18E-004	12
GO:0009199	ribonucleoside triphosphate metabolic process	P	4,25E-002	7,18E-004	12
GO:0009144	purine nucleoside triphosphate metabolic process	P	4,25E-002	7,18E-004	12

GO:0006873	cellular ion homeostasis	P	4,28E-002	7,43E-004	6
GO:0032535	regulation of cellular component size	P	4,28E-002	7,43E-004	6
GO:0090066	regulation of anatomical structure size	P	4,28E-002	7,43E-004	6
GO:0009141	nucleoside triphosphate metabolic process	P	4,28E-002	7,45E-004	12
GO:0008509	anion transmembrane transporter activity	F	4,32E-002	7,59E-004	7
GO:0009913	epidermal cell differentiation	P	4,42E-002	7,82E-004	10
GO:0008544	epidermis development	P	4,49E-002	7,99E-004	10
GO:0048522	positive regulation of cellular process	P	4,62E-002	8,29E-004	15
GO:0005984	disaccharide metabolic process	P	4,65E-002	8,46E-004	12
GO:0015672	monovalent inorganic cation transport	P	4,65E-002	8,54E-004	9
GO:0055044	symplast	C	4,65E-002	8,64E-004	14
GO:0009506	plasmodesma	C	4,65E-002	8,64E-004	14
GO:0044042	glucan metabolic process	P	4,65E-002	8,66E-004	15
GO:0048518	positive regulation of biological process	P	4,65E-002	8,71E-004	16
GO:0005911	cell-cell junction	C	4,69E-002	8,91E-004	14
GO:0071555	cell wall organization	P	4,69E-002	8,92E-004	12
GO:0030054	cell junction	C	4,69E-002	9,04E-004	14
GO:0009694	jasmonic acid metabolic process	P	4,69E-002	9,05E-004	5
GO:0055082	cellular chemical homeostasis	P	4,69E-002	9,09E-004	6
GO:0009814	defense response, incompatible interaction	P	4,70E-002	9,18E-004	9
GO:0010053	root epidermal cell differentiation	P	4,93E-002	9,70E-004	8
GO:0006955	immune response	P	4,94E-002	9,84E-004	13
GO:0005773	vacuole	C	4,94E-002	9,87E-004	18
GO:0008324	cation transmembrane transporter activity	F	4,94E-002	9,92E-004	12

Table S5 QTL detected in the '232' × '1392' strawberry population controlling the content of γ-decalactone and eQTL controlling the expression of *FaFAD1*, *FaFAH1* and *FaCYP1* based on Kruskal-Wallis (K-W) and interval mapping (IM). The position of the LOD peak (in cM) and the most closely associated marker locus is indicated. The estimated mean effect of the QTL (mu) associated with each of the genotypes (ac, ad, bc, bd) with phase type {00} and the data transformation used in the analysis is also indicated.

QTL	Year	K-W[a]	Location	Thr[b].	LOD	Position	Closest marker	R^2 (%)[c]	mu_ac{00}	mu_ad{00}	mu_bc{00}	mu_bd{00}	Transf.
γ-Decalactone	2007	****	III-2	14.7	26.02	72.493	BF45175cIII	84.4	0.017	0.802	0.029	2.795	-
γ-Decalactone	2008	****	III-2	9.3	21.52	69.644	BF45175cIII	92.8	0.023	1.392	0.032	3.571	-
γ-Decalactone	2009	****	III-2	15.3	29.75	70.644	BF45175cIII	89.9	0.029	2.800	0.032	1.401	-
eFaFAD1	2009	****	III-2	7.8	29.88	71.644	BF45175cIII	90	9.106	0.204	4.346	0.163	1/SQRT
eFaFAD1	2009	****	III-2	12.5	25.46	72.493	BF45175cIII	85	0.100	157.514	0.153	40.884	-
eFaFAH1	2009	****	III-2	4.5	8.17	64.644	BF45175cIII	55.4	-1.140	-0.278	-0.385	1.217	Ln
eFaCYP1	2009	****	III-1	4.7	13.86	5	ChFv233-218	55	-1.696	-1.236	0.762	1.310	Ln

[a]Significance level of Kruskal-Wallis test. *, p<0.005; **, p<0.001; ***, p<0.0005; ****, p<0.0001.
[b]LOD threshold.
[c]Percentage of the variance explained by the QTL.

Table S6 Expression of gene24414-v1.0-hybrid in each of the biological replicates by three different approaches

	H γ-DEC-1	H γ-DEC-2	H γ-DEC-3	N γ-DEC-1	N γ-DEC-2	N γ-DEC-3	H γ-DEC	N γ-DEC	Ratio	Log2ratio
Using reference genome (FPKM)	483.69	464.52	471.99	28.69	10.33	7.33	325.92*	11.41*	28.56	4.84
de novo assembly (FPKM)	497.45	463.4	485.01	29.49	9.78	6.83	481.95	15.37	31.36	4.97
qRT-PCR (relative expression)	88.44	113.77	71.01	6.99	1	0.99	91.07	2.99	30.43	4.93

*The final expression in each pool is lower than the average expression of the replicates because by mapping to the reference genome, cuffdiff uses the *F. vesca* predicted gene 24414 (which is longer than the *F. x ananassa* transcript) to quantify the expression as Fragments Per Kilobase of exon per Million fragments mapped (FPKM).
